# Chemical Composition and Antibacterial Activity Against Food-Borne Pathogens of Six Essential Oils from Plants in Northeastern Peru

**DOI:** 10.3390/ph19060951

**Published:** 2026-06-18

**Authors:** Laydy Mitsu Mena-Chacon, Krizia Pretell, Angel F. Huaman-Pilco, Yuriko Saavedra, Aline Camila Caetano, Diner Mori-Mestanza, Robin Oblitas-Delgado, Carlos A. Amasifuen-Guerra, Rocio Jara-Vilca, Roberth Esteve Iliquin-Fernandez, Segundo Chávez-Quintana

**Affiliations:** 1 Escuela de Posgrado, Programa Doctoral en Ciencias para el Desarrollo Sustentable, Facultad de Ingeniería Zootecnista, Agronegocios, Biotecnología y Ciencias de Datos, Universidad Nacional Toribio Rodríguez de Mendoza de Amazonas, Chachapoyas 01001, Peru; 2 Grupo de Investigación en Biopesticidas y Bioalternativas para la Protección Vegetal (BIOPEST), Instituto de Investigación para el Desarrollo Sustentable de Ceja de Selva, Universidad Nacional Toribio Rodríguez de Mendoza de Amazonas, Chachapoyas 01001, Peru; 3 Inca Biotec S.A.C., Jr. Filipinas 212, Tumbes 24000, Peru; 4 Grupo de Investigación en Hongos y Deterioro de Alimentos (GIHDA), Instituto de Investigación para el Desarrollo Sustentable de Ceja de Selva, Universidad Nacional Toribio Rodríguez de Mendoza de Amazonas, Chachapoyas 01001, Peru; 5 Instituto de Investigación en Forestería y Ecosistemas Tropicales (INIFET), Universidad Nacional Toribio Rodríguez de Mendoza de Amazonas (UNTRM), Chachapoyas 01001, Peru; 6 Instituto de Investigación para el Desarrollo Sustentable de Ceja de Selva, Universidad Nacional Toribio Rodríguez de Mendoza de Amazonas, Chachapoyas 01001, Peru

**Keywords:** chemotype variation, dose–response, food-borne pathogens, natural antimicrobials, multivariate analysis, natural preservatives

## Abstract

**Background:** Essential oils (EOs) are promising natural antimicrobials against food-borne pathogens, yet their efficacy depends on complex chemical profiles that vary by species and origin. The evaluation of underexplored aromatic plants from the Peruvian Amazon may reveal novel bioactive agents. **Methods:** We chemically characterized six EOs from *Aloysia citrodora*, *Arracacia xanthorrhiza* (two cultivars), *Baccharis genistelloides*, *Piper acutifolium*, and *Piper lanceifolium* using GC-MS and assessed their antibacterial activity against *Escherichia coli* (ATCC 25922), *Salmonella enterica* (ATCC 14028), *Enterococcus faecalis* (ATCC 29212), and *Staphylococcus aureus* (ATCC 49476). **Results:** EOs of *Aloysia citrodora* and *Arracacia xanthorrhiza* cv. Yellow exhibited the strongest inhibition, effective against both Gram-positive and Gram-negative bacteria, potentially associated with higher relative abundances of oxygenated monoterpenes and aliphatic aldehydes. Dose–response analysis supported their superior antibacterial potency, with the lowest LD_50_ values observed for these oils. Oils rich in sesquiterpenes showed lower activity. **Conclusions:** These findings underscore the importance of EO chemical composition for antibacterial potency and suggest that select Amazonian EOs have potential as natural preservatives for food safety applications.

## 1. Introduction

Food-borne diseases are an urgent and growing global public health concern. According to the World Health Organization (WHO), approximately 600 million people suffer from food-borne illnesses annually, resulting in an estimated 420,000 deaths worldwide. Children under five years of age account for nearly 30% of these deaths (approximately 125,000), despite representing only about 9% of the global population [1,2]. In addition to their health impacts, food-borne diseases generate substantial economic burdens on healthcare systems and the food industry. Dangerous pathogens such as Salmonella enterica, Escherichia coli, Staphylococcus aureus, and Enterococcus faecalis are routinely associated with food-borne outbreaks. Antimicrobial resistance is rapidly increasing among these microorganisms. This makes it imperative to find safe, effective, and sustainable alternatives to conventional antibiotics [3,4].

In this context, plant essential oils (EOs) have attracted considerable attention as natural antimicrobial agents. These oils are complex mixtures of volatile secondary metabolites. Their main components are terpenes (monoterpenes and sesquiterpenes), terpenoids, and phenylpropanoids. EOs exhibit a broad spectrum of biological activities. These include antibacterial, antioxidant, and anti-inflammatory properties [5,6,7,8]. Their antimicrobial activity is attributed to multiple mechanisms. These include disruption of cell membranes, leakage of intracellular components, inhibition of enzymatic systems, and interference with energy metabolism [3,9]. Importantly, the antimicrobial activity of essential oils often results from synergistic interactions among multiple constituents, rather than a single dominant compound [7,10,11].

Despite extensive research on EOs, comparing their antibacterial efficacy across studies is challenging. This is due to variations in extraction methods, chemical composition, bacterial strains, and experimental conditions [3,7,12]. Furthermore, the effectiveness of EOs is strongly influenced by their chemical profile. Such profiles can vary significantly depending on plant species, geographic origin, environmental conditions, and even intra-species factors such as cultivar or seasonality [10,13,14]. This chemical variability underscores the importance of detailed compositional analysis for proper interpretation of biological activity. Gas chromatography–mass spectrometry (GC–MS) remains the gold standard for such characterization. It enables accurate identification of volatile compounds and facilitates the exploration of structure–activity relationships [15].

From an applied perspective, the increasing demand for clean-label foods and natural preservatives has further stimulated interest in EOs as potential antimicrobial additives [16,17,18]. Several EO constituents, including citral, geraniol, linalool, and limonene, have been reported to show antibacterial activity in vitro against major food-borne pathogens [9,19]. These findings support their potential application in food preservation systems. In particular, the antimicrobial activity of *Aloysia citrodora* is primarily attributed to citral and limonene. These compounds are considered key contributors to its bioactivity [20,21].

The Peruvian Amazon represents a biodiversity hotspot with a wide variety of aromatic plant species. Many of these are traditionally used for medicinal and ethnobotanical purposes [22,23,24]. Nevertheless, many of these species remain poorly studied with respect to their chemical composition and biological activity. The species evaluated in the present study were selected because of their distinctive aromatic properties and their ethnobotanical importance among local communities in northeastern Peru. Additionally, they represent taxonomically diverse plant groups whose essential oils remain comparatively underexplored as potential antibacterial agents against food-borne pathogens. *Arracacia xanthorrhiza*, for example, is widely cultivated for its edible roots [25]. However, limited attention has been paid to the potential of its aerial parts, and previous reports on its EO composition are scarce and lack cultivar-specific information [26]. *Baccharis genistelloides* is widely recognized in South American traditional medicine. However, its biological properties have often been studied in pharmacological rather than food-safety contexts [27,28,29].

Species of the genus Piper are also of particular interest due to their broad ethnomedicinal use and their recognized potential as sources of bioactive compounds with antioxidant and antimicrobial properties. For *Piper lanceifolium*, a study from Ecuador described an EO rich in safrole. It reported moderate antibacterial activity against Klebsiella pneumoniae [30,31]. In the case of *Piper acutifolium* from Peru, recent work characterized an EO dominated by *α*-phellandrene, *β*-myrcene, and *β*-phellandrene. However, that study focused mainly on antioxidant and phytotoxic properties rather than on food-borne bacteria [23,32]. These reports highlight the biological potential of *Piper* species. They also reveal the lack of comparative studies integrating chemical profiling and antibacterial evaluation against relevant food-borne pathogens.

In this context, the present study aimed to characterize the chemical composition of six essential oils obtained from *Aloysia citrodora*, *Arracacia xanthorrhiza* cv. Yellow, *A. xanthorrhiza* cv. Purple, *Baccharis genistelloides*, *Piper acutifolium*, and *Piper lanceifolium* collected in the Peruvian Amazon using GC-MS; evaluate their antibacterial activity against major food-borne pathogens; and explore potential associations between chemical composition and antibacterial activity through multivariate analysis. The findings of the present study highlight chemical diversity among these native aromatic species and reveal distinct antibacterial profiles for several oils, underscoring their potential as sources of natural antimicrobial agents.

## 2. Results

### 2.1. Chemical Composition of Essential Oils

Extraction yields ranged from 0.52 ± 0.03% to 1.36 ± 0.04% across the species analyzed, with values of 0.87 ± 0.02% for *A. citrodora*, 0.63 ± 0.01% and 0.62 ± 0.02% for *A. xanthorrhiza* cv. Yellow and cv. Purple, 0.52 ± 0.03% for *B. genistelloides*, and 1.36 ± 0.04% and 1.05 ± 0.05% for *P. acutifolium* and *P. lanceifolium*, respectively.

**Figure 1:**
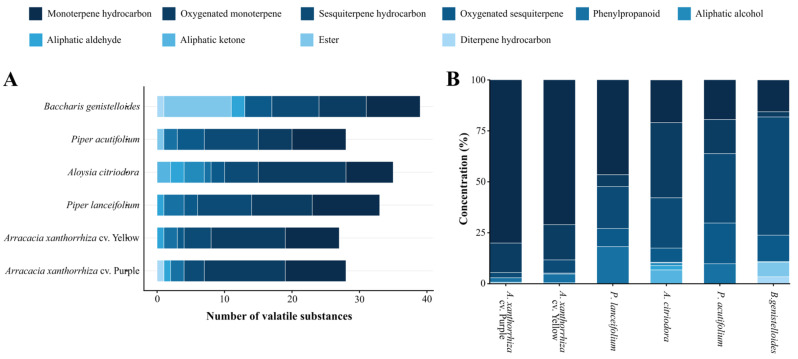
GC-MS characterization of six essential oils: (**A**) Distribution of volatile compounds according to their chemical families. (**B**) Relative abundance (%) of the main chemical families identified in each essential oil.

The GC–MS analysis allowed the identification of a total of 34, 24, 26, 39, 27, and 31 compounds in the EOs of *A. citrodora*, *A. xanthorrhiza* cv. Yellow, *A. xanthorrhiza* cv. Purple, *B. genistelloides*, *P. acutifolium*, and *P. lanceifolium*, respectively (Supplementary Table S8; Figure 1).

The EO of *A. citrodora* was mainly characterized by limonene (17.33 ± 0.18%), (R)-citronellol (15.06 ± 0.01%), and cuparene (10.11 ± 0.07%), followed by *β*-caryophyllene (8.09 ± 0.11%) and sulcatone (5.53 ± 0.11%) (Table 1). This profile was characterized by a predominance of oxygenated monoterpenes and sesquiterpene hydrocarbons (Figure 1).

In *A. xanthorrhiza* cv. Yellow, trans-*β*-ocimene (30.96 ± 0.44%), sabinene (21.46 ± 0.04%), and *β*-pinene (16.21 ± 0.01%), with additional contributions from linalool (5.55 ± 0.35%) and germacrene D (3.73 ± 0.72%), revealing a profile strongly dominated by monoterpene hydrocarbons. Similarly, oil of cv. Purple contained high levels of *β*-ocimene (30.50 ± 1.13%), sabinene (20.94 ± 0.48%), and *β*-pinene (14.16 ± 0.39%), along with trans-*β*-ocimene (10.15 ± 0.12%) and linalool (8.47 ± 0.03%), confirming a monoterpene-rich profile comparable to cv. Yellow (Table 1; Figure 1).

The EO of *B. genistelloides* was predominantly composed of sesquiterpene hydrocarbons, with *β*-caryophyllene (24.92 ± 0.46%) and *γ*-muurolene (13.30 ± 0.10%) as the major constituents. Other components included oxygenated sesquiterpenes, such as *α*-cadinol (7.67 ± 0.08%), and minor amounts of monoterpenes, such as limonene (6.82 ± 0.10%) (Table 1). This profile reflects a sesquiterpene-dominated composition (Figure 1).

In *P. acutifolium*, the main constituents were linalool (15.37 ± 0.52%), trans-*β*-ocimene (12.63 ± 0.60%), and epi-cubebol (10.16 ± 0.44%), followed by myristicin (9.41 ± 0.06%), *δ*-cadinene (8.79 ± 0.19%), and *β*-caryophyllene (8.12 ± 0.03%). This suggests a mixed profile of oxygenated monoterpenes and sesquiterpenes. Finally, the EO of *P. lanceifolium* was characterized by limonene (14.98 ± 0.01%), apiol (14.94 ± 0.35%), and *β*-caryophyllene (12.30 ± 0.04%), with additional *γ*-terpinene (9.27 ± 0.07%) and *β*-pinene (8.77 ± 0.16%) (Table 1), indicating the coexistence of monoterpene hydrocarbons, phenylpropanoids, and sesquiterpenes (Figure 1).

Given that only compounds with high match factors (≥80) and good agreement between experimental and library retention indices (ΔRI ≤ 20) were retained from raw data [33,34], the possibility of contamination or artifacts is minimized. Furthermore, although thermal transformations may occur during steam distillation [35], the major oxygenated monoterpenes identified in the present study, including linalool, citronellol, geraniol, nerol, and α-terpineol, have been previously reported as natural constituents of *Arracacia* spp. and several *Piper* species obtained through different extraction procedures [26,36,37]. Therefore, their occurrence is unlikely to be exclusively attributed to oxidation during extraction and more likely reflects intrinsic biosynthetic characteristics of the evaluated species.

### 2.2. Antibacterial Activity

The antibacterial inhibition varied significantly (*p* < 0.0001) among EOs, concentrations, and their interaction for all bacterial strains (Table 2; Supplementary Table S2). These results suggest that both the chemical nature of the EO and the applied concentration strongly influenced antibacterial activity. Detailed Tukey comparisons among EOs and doses are provided in Supplementary Tables S3 and S4.

**Figure 2:**
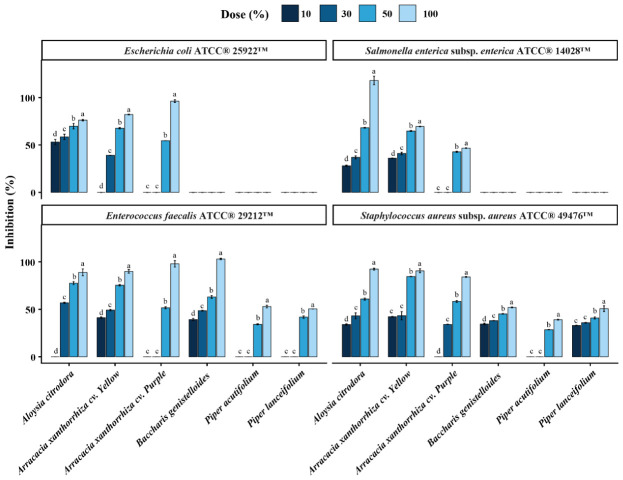
Dose-dependent antibacterial inhibition (%) of essential oils against four food-borne bacterial strains: *Escherichia coli* (ATCC^®^ 25922™), *Salmonella enterica* subsp. *enterica* (ATCC^®^ 14028™), *Enterococcus faecalis* (ATCC^®^ 29212™), and *Staphylococcus aureus* subsp. *aureus* (ATCC^®^ 49476™). Essential oils were evaluated at concentrations of 10, 30, 50, and 100% (*v*/*v* in DMSO). Bars represent mean values ± standard deviation (*n* = 3). Different letters indicate significant differences (*p* < 0.05) among concentrations of individual essential oils according to Tukey’s HSD test.

Across Gram-negative bacteria (*E. coli* and *S. enterica*), *A. citrodora* exhibited the highest inhibition percentages regardless of the concentration applied, followed by *A. xanthorrhiza* cv. Yellow and cv. Purple. According to the main-effect comparisons presented in Table S3, mean inhibition values reached 64.46% and 47.24% against *E. coli*, and 62.87% and 52.88% against *S. enterica*, respectively (*p* < 0.0001). In contrast, *A. xanthorrhiza* cv. Purple showed lower mean inhibition levels, with values of 37.72% against *E. coli* and 22.38% against *S. enterica* (Table S3). EOs from *P. lanceifolium*, *P. acutifolium*, and *B. genistelloides* did not show detectable antibacterial activity against Gram-negative bacteria under the tested conditions (Figure 2).

Figure 2 presents the complete dose-dependent responses for each essential oil–bacteria combination, whereas Table S3 summarizes the overall mean effects of essential oils independently of dose. Values above 100% in Figure 2 indicate that the inhibition zone produced by the essential oil exceeded that of the corresponding positive antibiotic control under the experimental conditions evaluated. This was observed for *A. citrodora* at 100% against *S. enterica* and for *B. genistelloides* at 100% against *E. faecalis*.

For Gram-positive bacteria, different patterns were observed. Against *E. faecalis*, the highest inhibition was recorded for *A. xanthorrhiza* cv. Yellow and *B. genistelloides*, which did not differ significantly (*p* > 0.05) from each other, with inhibition values of 63.95% and 63.52%, respectively, relative to the positive control vancomycin (30 µg) (Table S3). In the case of *S. aureus*, *A. xanthorrhiza* cv. Yellow showed the strongest antibacterial activity (65.20%), followed by *A. citrodora* (57.58%) (Table S3). In both Gram-positive species, EOs from the genus *Piper* exhibited the lowest antibacterial activity (Figure 2).

The results indicate that antibacterial activity varied markedly among EOs and bacterial species, with *A. citrodora* and *A. xanthorrhiza* cv. Yellow showing the most consistent inhibitory effects (Table S3; Figure 2).

All fitted models were highly significant (*p* < 0.0001), with R^2^ ranging from 0.72 to 0.99. RMSE values ranged between 1.95 and 19.11, indicating an overall adequate fit of the model to the experimental data, although with varying precision depending on the EO–bacteria combination (Table 3). Consistent with the observed inhibition patterns, the lowest LD50 values were estimated for *A. citrodora* against *E. coli*, with an LD_50_ of 8.97 ± 1.66%. This was followed by *A. xanthorrhiza* cv. Yellow, which showed strong inhibitory effects against *S. aureus* (18.43 ± 3.99%) and *E. faecalis* (18.59 ± 2.66%) (Table 3), suggesting comparatively stronger antibacterial activity within the evaluated concentration range.

In some EO–bacteria combinations, estimated LD_50_ values exceeded 100%, indicating that 50% inhibition was not reached within the concentration range experimentally evaluated. This was particularly observed for *Piper* species against *S. aureus* (Table 3). Therefore, these values should be interpreted as extrapolated estimates reflecting relatively low antibacterial potency under the tested conditions. Overall, lower LD_50_ values were associated with stronger antibacterial responses within the evaluated concentration range and supported the observation that *A. citrodora* and *A. xanthorrhiza* cv. Yellow exhibited the highest antibacterial activity among the evaluated EOs.

### 2.3. Multivariate Analysis

**Figure 3:**
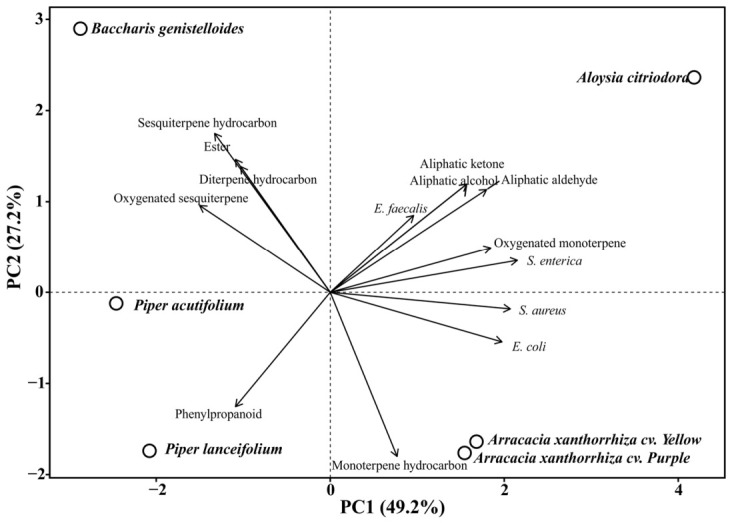
Principal component analysis (PCA) biplot integrating chemical families and antibacterial activity of essential oils from northeastern Peru. PCA biplot showing the association patterns between major chemical families identified by GC–MS and antibacterial activity against four food-borne bacterial strains. PC1 and PC2 explained 49.2% and 27.2% of the total variance, respectively. Oils enriched in oxygenated monoterpenes and aliphatic compounds were positioned closer to higher antibacterial activity, whereas sesquiterpene-rich and phenylpropanoid-dominated profiles were associated with comparatively lower antibacterial performance under the evaluated conditions.

Principal component analysis (PCA) was performed to explore multivariate association patterns between the chemical families identified in the essential oils and their antibacterial activity against the evaluated food-borne bacteria (Figure 3). The first two principal components explained 76.4% of the total variance, with PC1 and PC2 accounting for 49.2% and 27.2%, respectively (Table S5).

PC1 mainly separated essential oils according to antibacterial performance and chemical composition. Positive PC1 values were strongly associated with antibacterial activity against *S. enterica*, *S. aureus*, and *E. coli*, which showed some of the highest contributions to this component (13.9%, 12.9%, and 11.7%, respectively), together with oxygenated monoterpenes (10.2%) and aliphatic aldehydes (9.7%) (Tables S5 and S6). In contrast, negative PC1 values were associated with sesquiterpene hydrocarbons, oxygenated sesquiterpenes, phenylpropanoids, and lower antibacterial activity.

Within the ordination space, *A. citrodora* was clearly separated from the other oils and positioned in the positive region of PC1 and PC2, closely associated with oxygenated monoterpenes and antibacterial activity against Gram-negative bacteria, particularly *S. enterica* and *E. coli* (Figure 3; Table S7). *A. xanthorrhiza* cv. Yellow and cv. Purple were also positioned on the positive side of PC1, reflecting their relatively high antibacterial performance and association with monoterpene hydrocarbon-rich profiles.

Conversely, *P. lanceifolium*, *P. acutifolium*, and *B. genistelloides* were located on the negative side of PC1 and were more closely associated with sesquiterpene-rich and phenylpropanoid-dominated compositions (Figure 3). These oils were positioned opposite to most antibacterial activity vectors, consistent with their comparatively lower antibacterial performance under the evaluated conditions.

PC2 was mainly influenced by monoterpene hydrocarbons (17.6%), sesquiterpene hydrocarbons (16.6%), esters (11.7%), and diterpene hydrocarbons (10.4%) (Tables S5 and S6), contributing to the differentiation of *B. genistelloides* and the two *Arracacia* EOs. In addition, *E. faecalis* showed a distinct loading pattern compared with the other bacterial variables, suggesting some degree of differential susceptibility among the evaluated bacterial strains.

**Figure 4:**
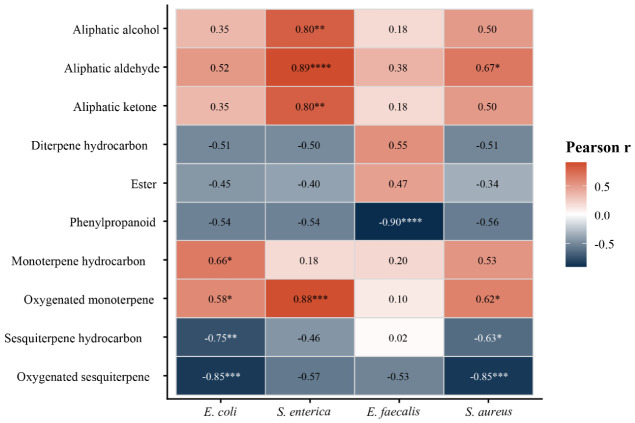
Pearson correlation between chemical families identified by GC–MS and antibacterial inhibition of essential oils against four bacterial strains. Heatmap showing Pearson correlation coefficients (r) between the relative abundance of major chemical families and antibacterial inhibition against *Escherichia coli* ATCC^®^ 25922™, *Salmonella enterica* subsp. *enterica* ATCC^®^ 14028™, *Enterococcus faecalis* ATCC^®^ 29212™, and *Staphylococcus aureus* subsp. *aureus* ATCC^®^ 49476™. Asterisks indicate significant correlations (* *p* < 0.05, ** *p* < 0.01, *** *p* < 0.001, **** *p* < 0.0001).

Pearson correlation analysis revealed that antibacterial activity was mainly associated with the relative abundance of specific oxygenated monoterpenes and aliphatic aldehydes in the EOs (Figure 4). In particular, strong positive correlations were observed between oxygenated monoterpenes and antibacterial inhibition against *S. enterica* (r = 0.88, *p* < 0.001), *E. coli* (r = 0.58, *p* < 0.05), and *S. aureus* (r = 0.62, *p* < 0.05). Similarly, aliphatic aldehydes showed a strong positive association with antibacterial activity, especially against *S. enterica* (r = 0.89, *p* < 0.0001) and *S. aureus* (r = 0.67, *p* < 0.05). Monoterpene hydrocarbons were also positively correlated with antibacterial inhibition against *E. coli* (r = 0.66, *p* < 0.05).

In contrast, sesquiterpene-rich fractions were negatively associated with antibacterial activity (Figure 4). Oxygenated sesquiterpenes showed strong negative correlations with inhibition of *E. coli* (r = −0.85, *p* < 0.001) and *S. aureus* (r = −0.85, *p* < 0.001), while sesquiterpene hydrocarbons were negatively correlated with inhibition of *E. coli* (r = −0.75, *p* < 0.01) and *S. aureus* (r = −0.63, *p* < 0.05). For E. faecalis, antibacterial activity was strongly negatively correlated with phenylpropanoids (r = −0.90, *p* < 0.0001).

PCA and Pearson correlation analyses revealed consistent exploratory association patterns between essential oil composition and antibacterial activity (Figure 3 and Figure 4). EOs characterized by higher relative abundances of oxygenated monoterpenes, aliphatic aldehydes, and selected aliphatic compounds tended to group closer to antibacterial inhibition variables, particularly against *S. enterica*, *E. coli*, and *S. aureus*. In contrast, sesquiterpene-rich and phenylpropanoid-dominated profiles tended to be positioned opposite to most antibacterial activity variables in the multivariate space.

The EOs analyzed exhibited distinct compositional profiles (Table 1). Oxygenated monoterpenes were mainly represented by compounds such as (R)-citronellol, geraniol, nerol, neral, and *α*-terpineol in *A. citrodora*, as well as linalool in *P. acutifolium* and *A. xanthorrhiza*. Monoterpene hydrocarbons were dominated by trans-*β*-ocimene, sabinene, *β*-pinene, and limonene in *Arracacia* and *Piper* species. In addition, several aliphatic compounds, including 1-octen-3-ol, nonanal, octanal, sulcatone, and 2-undecanone, although present at lower relative abundance, showed exploratory association patterns with antibacterial activity in the multivariate analyses.

Nevertheless, these results should be interpreted cautiously, as multivariate and correlation analyses do not establish direct causality. The antibacterial activity of EOs likely depends on complex synergistic or additive interactions among multiple constituents rather than on the effect of a single dominant compound.

## 3. Discussion

The antibacterial activity of the EOs evaluated here depended strongly on both plant species and bacterial strain, as well as on the chemical profile of each oil. Across factorial ANOVA, dose–response modeling, and correlation analysis, *A. citrodora* and *A. xanthorrhiza* cv. Yellow consistently showed the strongest antibacterial performance, whereas *P. lanceifolium* and *P. acutifolium* were the least effective. These results highlight compositional differences among the evaluated oils that may help explain the variability observed in antibacterial activity.

A marked difference was observed between Gram-negative and Gram-positive bacteria. Under the present conditions, inhibition of E. coli and S. enterica was mainly restricted to *A. citrodora* and the two *Arracacia* oils. *B. genistelloides* and both *Piper* oils showed no detectable activity. In contrast, Gram-positive bacteria, especially E. faecalis and S. aureus, were inhibited by a broader range of oils. This pattern aligns with the widely reported tendency of EOs to be more effective against Gram-positive than Gram-negative bacteria.

However, this trend is not universal and depends on both the target microorganism and oil composition [3,9,38,39]. The higher tolerance of Gram-negative bacteria is commonly linked to the outer membrane, which restricts the diffusion of hydrophobic compounds [9,11,40]. In addition, Gram-negative bacteria possess lipopolysaccharide-rich outer membranes, efflux pump systems, and detoxification mechanisms that may further reduce the intracellular accumulation and effectiveness of hydrophobic EO constituents [41,42]. However, the strong activity of *A. citrodora* against both *E. coli* and *S. enterica* shows that this barrier is not absolute. Bacterial susceptibility is better understood as the result of interactions between envelope structure and oil chemistry, rather than Gram classification alone [19,21,43]. Similar patterns of higher susceptibility of Gram-positive bacteria and reduced activity against Gram-negative species have been previously reported for several terpene-rich essential oils, particularly those dominated by sesquiterpene fractions [4,44,45,46].

This interpretation was reinforced by the dose–response analysis. The lowest LD_50_ values were obtained for *A. citrodora* against *E. coli* and for *A. xanthorrhiza* cv. Yellow against both Gram-positive strains. These results suggest that these EOs achieved inhibitory effects at comparatively low concentrations. In this sense, LD_50_ was particularly useful because it provided a complementary, model-derived indicator of relative antibacterial potency beyond inhibition percentages alone [5,43].

Nevertheless, some EO–bacteria combinations yielded estimated LD_50_ values above the experimentally evaluated concentration range (>100%), particularly for *Piper* oils against *S. aureus* (Table 3). This indicates comparatively weak antibacterial activity under the tested conditions, as 50% inhibition was not achieved within the concentration range evaluated (Figure 2). Therefore, these values should be interpreted as extrapolated estimates rather than direct experimental measurements. From a biological perspective, this reduced effectiveness may be related to the predominance of sesquiterpene-rich profiles or to lower abundances of oxygenated monoterpenes and aldehyde-associated compounds, which have been more frequently associated with antibacterial activity in previous studies [6,7,47]. However, these interpretations should be considered cautiously, as essential oil activity likely depends on complex synergistic interactions among multiple constituents.

The multivariate analyses further clarified the compositional patterns underlying the observed differences in antibacterial activity. EOs characterized by higher relative abundances of oxygenated monoterpenes and aliphatic aldehydes tended to group closer to antibacterial inhibition variables, particularly those associated with *E. coli*, *S. enterica*, and *S. aureus*. In contrast, sesquiterpene-rich and phenylpropanoid-dominated profiles were generally positioned opposite to most antibacterial activity variables in the multivariate space. Although these associations do not establish direct causality for individual compounds, they consistently suggest that monoterpene-related fractions may contribute more strongly to antibacterial performance than sesquiterpene-dominated profiles under the evaluated conditions. This is consistent with the known antimicrobial behavior of terpenes and terpenoids, which may affect membrane integrity, protein function, intracellular leakage, and energy metabolism [4,9,19].

These exploratory multivariate patterns are consistent with recent studies integrating PCA and correlation-based approaches to associate essential oil chemotypes with antimicrobial activity [48,49]. In particular, compounds such as citronellol, geraniol, citral-related molecules, linalool, and α-terpineol have frequently been associated with increased membrane permeability, disruption of lipid bilayer organization, intracellular leakage, and interference with respiratory and enzymatic processes in bacteria [9,41,46]. In addition, aliphatic aldehydes and ketone-related compounds, even at relatively low abundance, may contribute to antibacterial effects because of their chemical reactivity and potential interactions with membrane-associated proteins and cellular redox systems [42,43]. Vasconcelos et al. [49] further demonstrated that structurally related oxygenated monoterpenes may exhibit distinct antibacterial and antibiofilm performances depending on their oxygenated functional groups, reinforcing the importance of compositional variability within terpene-rich oils.

A summary of the antibacterial activities previously reported for the major constituents identified in the present study is provided in Table 4. Collectively, these reports support the potential contribution of compounds such as limonene, citronellol, linalool, sabinene, β-pinene, trans-β-ocimene, β-caryophyllene, apiol, and myristicin to the antibacterial patterns observed herein, although synergistic interactions among constituents likely play a central role.

**Figure 5:**
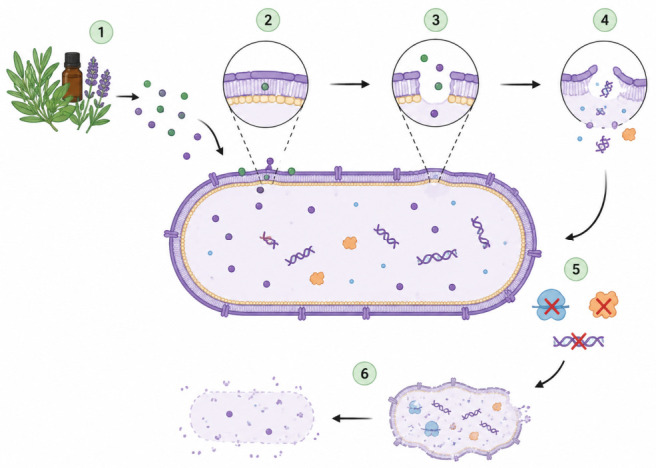
General mechanisms of antibacterial action of essential oil constituents against food-borne bacterial pathogens. Essential oil constituents interact with the bacterial cell envelope, promoting membrane destabilization and increased permeability. These alterations facilitate leakage of intracellular components, disrupt membrane-associated proteins and enzymatic systems, interfere with metabolic processes, and impair cellular homeostasis. The combined effects may ultimately result in growth inhibition and bacterial cell death. The antibacterial activity of essential oils is generally attributed to the additive and/or synergistic action of multiple constituents rather than to a single compound. (1) Release of essential oil constituents; (2) interaction with the bacterial membrane; (3) increased membrane permeability and structural disruption; (4) leakage of intracellular components; (5) impairment of essential cellular functions, including enzymatic and metabolic processes; (6) bacterial cell death.

The antibacterial activity of essential oils is generally attributed to multiple complementary mechanisms involving membrane disruption, increased permeability, leakage of intracellular constituents, and interference with essential cellular processes (Figure 5).

Similarly, the positive associations observed here between oxygenated monoterpenes, aliphatic aldehydes, and inhibition of *S. enterica* and *E. coli* agree with recent reports identifying these chemical classes as important contributors to antibacterial activity against food-borne pathogens [69,70]. In contrast, oils dominated by sesquiterpene hydrocarbons and oxygenated sesquiterpenes tended to exhibit comparatively lower antibacterial activity, particularly against Gram-negative bacteria. This may be partially related to differences in molecular size, volatility, polarity, and membrane diffusion capacity among terpene classes, although recent studies also emphasize that the overall antibacterial performance of essential oils likely depends on complex synergistic or additive interactions among multiple constituents rather than on isolated compounds [3,42,44,47,71].

Within this context, *A. citrodora* stands out as the clearest example of a chemically and biologically coherent profile. Its oil was rich in limonene and oxygenated monoterpenes, especially (R)-citronellol, geraniol, nerol, citral, and *α*-terpineol. All of these have been previously associated with antimicrobial activity in *A. citrodora* and related systems [13,20,72]. The strong activity observed here is therefore consistent with earlier reports. However, it extends them by demonstrating substantial inhibition of both Gram-negative and Gram-positive food-borne bacteria. The particularly low LD_50_ against *E. coli* is notable. Rather than attributing this response to a single constituent, the present results suggest the interpretation of *A. citrodora* as a bioactive monoterpene-rich mixture. Limonene, citral-related compounds, citronellol, geraniol, and other oxygenated monoterpenes likely contribute jointly to antibacterial action [4,9,21].

A second relevant finding concerns *A. xanthorrhiza* cv. Yellow. This oil is dominated by monoterpene hydrocarbons, including trans-*β*-ocimene, sabinene, and *β*-pinene. Yet, it showed one of the most consistent antibacterial responses, particularly against *E. faecalis* and *S. aureus*. This is notable because monoterpene hydrocarbons are often considered less active than oxygenated monoterpenes [6,46]. However, the presence of linalool and other oxygenated constituents suggests that the observed activity may depend on the balance among these compounds rather than on any single dominant fraction. Linalool has been repeatedly reported to exhibit antibacterial activity, especially against *Staphylococcus* spp. Its effect, though, may vary with assay conditions and test system [7,12,73]. The stronger performance of cv. Yellow compared with cv. Purple, despite its broadly similar profiles, further supports the idea that relatively subtle compositional differences, including minor constituents, may significantly influence biological activity. This interpretation is consistent with reports of intraspecific chemical variation associated with geography, season, year, or cultivar identity [13,24]. This finding adds value to the present study, given the limited cultivar-specific information available for *A. xanthorrhiza*.

In contrast, oils richer in sesquiterpene fractions, such as *B. genistelloides* and the two *Piper* species, were generally less active, particularly against Gram-negative bacteria. These oils contained recognized bioactive constituents, including *β*-caryophyllene, *α*-cadinol, cedrol, bisabolol, myristicin, and apiol [7,11,74]. However, their overall antibacterial performance was limited. The negative correlations observed for sesquiterpene hydrocarbons and oxygenated sesquiterpenes suggest that, in this experimental system, these fractions were not the main drivers of inhibition. This does not imply that sesquiterpenes are inactive, but within the compositional context of these oils, they were associated with lower activity than monoterpene-rich profiles [19,27,30]. The same applies to phenylpropanoids: although compounds like eugenol are known to disrupt membrane integrity and promote leakage of intracellular components, their impact depends on abundance, chemical context, and interactions within the oil matrix [5,9,47]. In this study, phenylpropanoids were not positively associated with antibacterial activity. This reinforces that the presence of a known bioactive compound does not necessarily predict the overall performance of a complex EO.

One of the most relevant outcomes of this study is that the chemical families most closely associated with antibacterial activity were not always the most abundant. Oxygenated monoterpenes and aliphatic aldehydes showed positive relationships with inhibition, even at relatively low abundance. This suggests that antibacterial effectiveness may depend more on the intrinsic bioactivity of specific constituents and their interactions than on quantitative dominance alone [9,39]. Minor constituents can enhance permeability or potentiate the activity of more reactive molecules [3,19]. The correlations observed here for oxygenated monoterpenes and aldehydes are particularly meaningful. These groups included compounds like citronellol, geraniol, citral-related molecules, and linalool, all of which have been linked to membrane disruption and metabolic impairment [4,7,47]. However, these associations should be interpreted with caution, since correlation analyses do not demonstrate causality, and mechanistic confirmation was beyond the scope of the present study.

From an applied perspective, the strong antibacterial performance of *A. citrodora* and *A. xanthorrhiza* cv. Yellow identifies them as promising candidates for the development of natural antimicrobial systems for food preservation. Some of these oils, particularly those from *Arracacia*, have not been widely valorized in this context. Nevertheless, the present findings were obtained under in vitro conditions, so direct extrapolation to food systems should be made with caution. EO efficacy can vary substantially depending on the food matrix, processing conditions, and delivery system. These include emulsions, coatings, and active packaging [12,74,75]. Future works should therefore assess these oils in more complex food models and explore formulation strategies such as encapsulation, emulsification, or combinations with other natural antimicrobials, including chitosan-based systems.

The present study indicates that the antibacterial activity of these underexplored essential oils was associated with differences in chemical composition, particularly with monoterpene-rich and oxygenated profiles. Within the evaluated set, *A. citrodora* and *A. xanthorrhiza* cv. Yellow exhibited the most consistent antibacterial performance, whereas sesquiterpene-rich oils were comparatively less effective under the tested conditions. These findings highlight the value of compositional profiling for identifying promising antibacterial patterns among underutilized aromatic plant resources and support their exploratory potential for future food preservation applications. Nevertheless, the present work should be interpreted as an exploratory in vitro screening study focused on identifying promising antibacterial profiles in underexplored Amazonian essential oils, rather than as a comprehensive mechanistic or pharmacological characterization.

From a taxonomic perspective, the strongest antibacterial activity was observed in species belonging to Verbenaceae (*A. citrodora*) and Apiaceae (*A. xanthorrhiza*), whereas the two Piperaceae species exhibited comparatively lower activity. Similar trends have been reported for other members of these families. Within Verbenaceae, several *Aloysia* species have demonstrated antibacterial activity against both Gram-positive and Gram-negative bacteria, including methicillin-resistant *S. aureus* [76,77]. Likewise, Apiaceae species producing monoterpene-rich EOs have shown promising antibacterial and antibiofilm properties. For example, *Daucus nebrodensis* EO, characterized by high proportions of sabinene and α-pinene, exhibited activity against both Gram-positive and Gram-negative pathogens [78]. In contrast, antibacterial activity within Piperaceae appears highly variable and strongly dependent on chemotype composition, with substantial interspecific and intraspecific variation reported among *Piper* species [79,80]. These observations suggest that antibacterial efficacy is more closely associated with phytochemical composition than with taxonomic affiliation alone, although certain plant families may exhibit characteristic chemical profiles that favor antimicrobial activity.

From an applied perspective, the strong antibacterial performance of *A. citrodora* and *A. xanthorrhiza* cv. Yellow identifies them as promising candidates for the development of natural antimicrobial systems for food preservation. Nevertheless, the concentrations evaluated in the present study were selected to characterize antibacterial potential under controlled in vitro conditions and should not be interpreted as concentrations directly applicable to food systems. Practical implementation will require efficacy validation in real food matrices, where interactions with food components may influence antimicrobial performance. In addition, factors such as sensory acceptability, concentration-dependent effects on food quality, potential toxicity, and regulatory requirements must be carefully considered before commercial application. Future studies should therefore evaluate these essential oils in more complex food models and determine MIC and MBC values, while also exploring formulation strategies, including encapsulation, active packaging systems, and combinations with other natural antimicrobials, and integrating microbiological, toxicological, and organoleptic assessments to determine their practical suitability for food preservation.

## 4. Materials and Methods

### 4.1. Plant Material and Essential Oil Extraction

Healthy and fully expanded plant material from six species (Table 5) was collected in September 2025 during the dry season in the Amazonas region, Peru (WGS84 coordinates detailed in Table 4). Species were selected based on their local abundance and ethnobotanical relevance in the Amazonas region. Taxonomic identification was performed by a specialist botanist, and voucher specimens were deposited at the KUÉLAP Herbarium of the National University Toribio Rodríguez de Mendoza de Amazonas (UNTRM). Voucher codes are provided in Table 5 to ensure traceability.

After collection, samples were cleaned and air-dried in the shade at ambient temperature for three days. EOs were obtained by hydro distillation using a Clevenger-type apparatus (Tecnal TE-2761, Tecnal Equipamentos Científicos, Piracicaba, Brazil) for 3 h, following standard procedures for aromatic plants [15,39,43]. Distillation was performed in triplicate using independent batches in order to determine the extraction yield. The recovered EO was separated from the aqueous phase, dried over anhydrous sodium sulfate to remove residual moisture, and stored in amber glass vials at 4 °C until further chemical and biological analyses.

### 4.2. Chemical Analysis by GC-MS

The chemical composition of EOs was determined by gas chromatography coupled to mass spectrometry (GC–MS) using an Agilent 7890B GC system equipped with a 5977B mass selective detector (Agilent Technologies, Santa Clara, CA, USA). EOs were diluted in hexane (1:50, *v*/*v*), and 0.5 µL of the solution was injected in splitless mode. Chromatographic separation was carried out on a DB-5MS UI capillary column (60 m × 0.25 mm i.d. × 1.0 µm film thickness). Helium was used as carrier gas at a constant flow rate of 1.0 mL/min.

The injector, quadrupole, transfer line, and ion source temperatures were set at 220, 150, 240, and 280 °C, respectively. The oven temperature program was initially set at 60 °C, increased at 3 °C min^−1^ to 246 °C, and held for 15 min. Mass spectra were acquired in electron ionization mode (EI, 70 eV) in scan mode over a mass range of *m*/*z* 40–600. Compounds were initially identified by comparison of their mass spectra with the NIST 17 mass spectral library and by deconvolution using MassHunter Unknowns Analysis software. Linear retention indices (LRI) were calculated according to the Van den Dool and Kratz method using a homologous series of n-alkanes (C_8_–C_20_, ~40 mg/L each, in hexane, Supelco, Sigma-Aldrich, St. Louis, MO, USA) analyzed under the same chromatographic conditions and compared with literature data [81,82].

To ensure robust identification, only compounds showing a spectral similarity match ≥ 90% and a difference between experimental and literature retention indices |ΔRI| ≤ 40 were considered for final reporting [83]. Relative abundances were calculated as normalized peak areas (%).

Although the DB-5MS column employed is not specifically designed for the separation of structural or enantiomeric isomers, minor differences in analyte–stationary phase interactions may allow partial chromatographic resolution. However, isomeric compounds often exhibit highly similar mass spectra and only slight differences in retention indices. Therefore, compound identification based solely on GC–MS data should be considered tentative and interpreted with caution. Whenever possible, confirmation with authentic standards or complementary analytical techniques is recommended.

### 4.3. Bacterial Strains and Culture Conditions

The antibacterial activity of the EOs was evaluated using the agar disk diffusion assay, adapted from the Kirby–Bauer disk diffusion method for hydrophobic natural products [73,84,85]. Although agar diffusion assays do not provide minimum inhibitory concentration or minimum bactericidal concentration values, they are widely used as an initial screening approach to compare the relative antibacterial activity of EOs and other natural extracts under standardized experimental conditions [73,85,86,87]. The bacterial strains *Escherichia coli* (ATCC^®^ 25922™), *Salmonella enterica* subsp. *enterica serovar Typhimurium* (ATCC^®^ 14028™), *Enterococcus faecalis* (ATCC^®^ 29212™), and *Staphylococcus aureus* subsp. *aureus* (ATCC^®^ 49476™) were used in this study. Strains were cultured in nutrient broth at 37 °C for 18–24 h before testing.

The bacterial inoculum was adjusted to the turbidity equivalent of a 0.5 McFarland standard (1.3 × 10^8^ cel. mL^−1^) using a spectrophotometer (NanoDrop™, Thermo Fisher Scientific, Waltham, MA, USA). A volume of 100 µL of the standardized suspension was uniformly spread onto Mueller–Hinton agar plates. Sterile paper disks (6 mm diameter) were impregnated with 10 µL of EO at concentrations of 10, 30, 50, and 100% (*v*/*v* in DMSO) and placed on the inoculated agar surface. Positive controls included vancomycin (30 µg) for *E. faecalis*, amoxicillin–clavulanic acid (30 µg) for *E. coli*, and florfenicol (30 µg) for *S. enterica* and *S. aureus*. DMSO (100%) was used as a negative control in all assays and produced no detectable inhibition against any bacterial strain. These concentrations corresponded to commercially standardized antibiotic disks commonly used in routine antimicrobial susceptibility testing and adapted Kirby–Bauer assays [88].

Plates were incubated at 37 °C for 24 h. The diameters of complete inhibition zones (absence of visible growth) were measured in millimeters using a digital caliper. For comparative purposes, the inhibition percentage relative to the corresponding antibiotic control was calculated as:

(1) $$Inhibition(\%)=\frac{D_{treatment}}{D_{control}}\times 100$$

where *D_treatment_* represents the inhibition zone produced by the EO, and *D_control_* corresponds to that produced by the positive control.

Each treatment was performed in triplicate, and results were expressed as mean ± standard deviation.

### 4.4. Statistical Analysis

Antibacterial inhibition percentages relative to the corresponding antibiotic control were calculated for each treatment and analyzed separately for each bacterial strain. The effects of EOs, concentration, and their interaction (EO × concentration) on antibacterial inhibition were evaluated using a two-way factorial analysis of variance (ANOVA). When significant effects were detected, mean comparisons were performed using Tukey’s honestly significant difference (HSD) test at a significance level of *p* < 0.05. The assumptions of normality and homogeneity of variance were assessed using the Shapiro–Wilk and Levene tests, respectively.

Dose–response relationships were modeled using a log-logistic regression to estimate the lethal dose required to achieve 50% inhibition (LD_50_). Model performance was evaluated using the model significance (*p*), the coefficient of determination (R^2^), and the root mean square error (RMSE).

Principal component analysis (PCA) was performed to explore multivariate association patterns between the relative abundance of major chemical families identified by GC–MS and the antibacterial inhibition values obtained for each bacterial strain. The analysis was conducted using mean values for each essential oil, and variables were standardized prior to analysis. PCA scores, loadings, and variable contributions were extracted to aid interpretation of compositional and antibacterial activity patterns among essential oils. Biplots were generated to visualize the relationships between chemical composition and antibacterial activity variables.

Pearson correlation analysis was conducted to evaluate associations between the relative abundance of major chemical families identified by GC–MS and antibacterial inhibition at the highest tested concentration (100%) for each bacterial strain. Correlation coefficients (r) and significance levels were visualized using a correlation heatmap. All statistical analyses were performed in R (version 4.4.1) using the packages *dcr*, *FactoMineR*, *factoextra*, and *pheatmap*.

## 5. Conclusions

The present study provides comparative information on the chemical composition and antibacterial activity of six underexplored essential oils from the Peruvian Amazon. Among the evaluated oils, *Aloysia citrodora* and *Arracacia xanthorrhiza* cv. Yellow showed the most consistent antibacterial activity, including inhibitory effects against Gram-positive bacteria and, in the case of *A. citrodora*, also against Gram-negative food-borne bacteria at comparatively low concentrations. Multivariate analyses suggested exploratory association patterns between antibacterial activity and oils enriched in oxygenated monoterpenes and aliphatic aldehydes, whereas sesquiterpene-rich profiles tended to be associated with comparatively lower antibacterial performance under the tested conditions. Overall, these findings highlight the value of compositional profiling for identifying promising antibacterial patterns among underutilized Amazonian aromatic plants and reinforce the need for future mechanistic, formulation, and food-system validation studies.

## Figures and Tables

**Table 1:** Major volatile compounds (>1% relative abundance) identified in the essential oils by GC–MS analysis.

RT (min)	Compound Name	RA (%)	Formula	MF (%)	Experimental RI	Library RI
*Aloysia citrodora*						
21.77	Tricyclene	1.19 ± 0.06	C_10_H_16_	98.59	946	890
23.77	Sulcatone	5.53 ± 0.11	C_8_H_14_O	91.96	983	986
24.19	*β*-Myrcene	1.30 ± 0.04	C_10_H_16_	96.11	990	991
26.95	Limonene	17.33 ± 0.18	C_10_H_16_	98.07	1041	1018
27.72	5-Heptenal, 2,6-dimethyl-	1.27 ± 0.06	C_9_H_16_O	92.65	1055	1052
30.21	Linalool	1.06 ± 0.01	C_10_H_18_O	97.24	1101	1099
34.39	Neral	2.98 ± 0.00	C_10_H_16_O	96.85	1181	1184
35.82	*α*-Terpineol	1.97 ± 0.01	C_10_H_18_O	97.34	1209	1189
36.7	Citronellol	15.06 ± 0.01	C_10_H_20_O	97.9	1227	1220
36.84	Nerol	2.76 ± 0.02	C_10_H_18_O	98.34	1230	1228
37.93	Geraniol	3.26 ± 0.02	C_10_H_18_O	98.76	1252	1255
39.96	2-Undecanone	1.25 ± 0.01	C_11_H_22_O	92.51	1293	1294
43.82	Geranyl acetate	4.64 ± 0.04	C_12_H_20_O_2_	98.5	1375	1382
47.39	*β*-Caryophyllene	8.09 ± 0.11	C_15_H_24_	93.91	1455	1419
47.91	Geranyl propionate	2.87 ± 0.09	C_13_H_22_O_2_	92.81	1467	1475
49.17	Cuparene	10.11 ± 0.07	C_15_H_22_	96.27	1496	1483
49.7	Germacrene D	3.28 ± 0.08	C_15_H_24_	98.26	1509	1495
50.35	*δ*-Elemene	2.28 ± 0.01	C_15_H_24_	95.1	1524	1514
52.19	Nerolidol	2.95 ± 0.09	C_15_H_26_O	97.21	1569	1564
54.49	Caryophyllene oxide	4.03 ± 0.16	C_15_H_24_O	94.79	1626	1581
*Arracacia xanthorrhiza* cv. Yellow						
21.1	*α*-Pinene	1.24 ± 0.02	C_10_H_16_	94.71	934	937
23.83	Sabinene	21.46 ± 0.04	C_10_H_16_	95.26	984	974
24.39	*β*-Pinene	16.21 ± 0.01	C_10_H_16_	94.58	994	979
26.63	*p*-Cymene	2.72 ± 0.00	C_10_H_14_	97.31	1035	1025
27.33	trans-*β*-Ocimene	30.96 ± 0.44	C_10_H_16_	96.5	1048	1049
30.21	Linalool	5.55 ± 0.35	C_10_H_18_O	97.47	1101	1099
34.49	Pinocarvone	1.10 ± 0.02	C_10_H_14_O	95.36	1183	1171
35.21	*α*-Terpineol	1.14 ± 0.06	C_10_H_18_O	94.77	1197	1182
35.8	Estragole	2.89 ± 0.15	C_10_H_12_O	97.94	1209	1196
36.89	Carveol	1.68 ± 0.06	C_10_H_16_O	98.08	1231	1229
38.31	Carvone	1.27 ± 0.09	C_10_H_14_O	97.32	1259	1242
40.71	trans-Pinocarvyl acetate	1.19 ± 0.11	C_12_H_18_O_2_	95.79	1309	1297
42.07	trans-Carveyl acetate	1.01 ± 0.16	C_12_H_18_O_2_	97	1338	1337
45.01	Methyleugenol	1.36 ± 0.28	C_11_H_14_O_2_	93.52	1401	1402
45.61	*β*-Bourbonene	1.10 ± 0.19	C_15_H_24_	95.05	1415	1384
47.37	*β*-Caryophyllene	1.29 ± 0.23	C_15_H_24_	96.54	1455	1419
49.95	Germacrene D	3.73 ± 0.72	C_15_H_24_	94.34	1515	1481
*Arracacia xanthorrhiza* cv. Purple						
23.83	Sabinene	20.94 ± 0.48	C_10_H_16_	95.23	984	974
24.39	*β*-Pinene	14.16 ± 0.39	C_10_H_16_	94.45	994	979
26.63	*p*-Cymene	2.04 ± 0.04	C_10_H_14_	97.41	1035	1025
26.79	trans-*β*-Ocimene	10.15 ± 0.12	C_10_H_16_	98.84	1038	1049
27.34	*β*-Ocimene	30.05 ± 1.13	C_10_H_16_	98.6	1048	1037
28.4	*γ*-Terpinene	1.86 ± 0.13	C_10_H_16_	98.55	1068	1060
30.21	Linalool	8.47 ± 0.03	C_10_H_18_O	97.47	1101	1099
35.21	Terpinen-4-ol	2.04 ± 0.03	C_10_H_18_O	93.33	1197	1182
35.8	Estragole	1.93 ± 0.00	C_10_H_12_O	98.95	1209	1196
47.37	*β*-Caryophyllene	1.45 ± 0.01	C_15_H_24_	98.18	1455	1419
*Baccharis genistelloides*						
24.2	*β*-Myrcene	1.62 ± 0.01	C_10_H_16_	94.09	990	991
25.67	*α*-Phellandrene	1.23 ± 0.00	C_10_H_16_	97.64	1017	1005
26.65	trans-*β*-Ocimene	1.43 ± 0.00	C_10_H_16_	97.79	1035	1049
26.93	Limonene	6.82 ± 0.10	C_10_H_16_	98.25	1041	1018
27.16	*β*-Phellandrene	3.05 ± 0.01	C_10_H_16_	94.9	1045	1031
37.85	2-Methylbutyl hexanoate	2.17 ± 0.01	C_11_H_22_O_2_	97.45	1250	1247
38.23	Hexanoic acid, 4-pentenyl ester	1.11 ± 0.03	C_11_H_20_O_2_	93.84	1258	1272
44.79	Ylangene	1.21 ± 0.09	C_15_H_24_	94.76	1396	1372
45.08	Copaene	5.02 ± 0.11	C_15_H_24_	96.66	1403	1376
47.4	*β*-Caryophyllene	24.92 ± 0.46	C_15_H_24_	99.1	1456	1419
49.42	*γ*-Muurolene	13.3 ± 0.10	C_15_H_24_	97.61	1502	1477
49.64	Naphthalene, 1,2,4a,5,6,8a-hexahydro-4,7-dimethyl-1-(1-methylethyl)-	8.21 ± 0.19	C_15_H_24_	95.01	1507	1485
50.33	*α*-Muurolene	4.64 ± 0.07	C_15_H_24_	97.04	1524	1499
55.8	Isospathulenol	1.41 ± 0.03	C_15_H_24_O	90.45	1659	1638
56.36	*τ*-Cadinol	2.88 ± 0.07	C_15_H_26_O	97.14	1673	1640
56.95	*α*-Cadinol	7.67 ± 0.08	C_15_H_26_O	97.52	1688	1653
61.02	Benzyl Benzoate	1.55 ± 0.09	C_14_H_12_O_2_	98.9	1798	1762
62.51	Neophytadiene	3.41 ± 0.33	C_20_H_38_	95.18	1837	1837
*Piper acutifolium*						
26.66	trans-*β*-Ocimene	12.63 ± 0.6	C_10_H_16_	98.56	1036	1049
28.4	*γ*-Terpinene	1.92 ± 0.00	C_10_H_16_	94.3	1068	1060
29.99	Terpinolene	3.04 ± 0.12	C_10_H_16_	96.63	1097	1088
30.22	Linalool	15.37 ± 0.52	C_10_H_18_O	97.93	1101	1099
45.08	Copaene	2.86 ± 0.01	C_15_H_24_	97.09	1403	1376
47.38	*β*-Caryophyllene	8.12 ± 0.03	C_15_H_24_	99.07	1455	1419
48.96	Humulene	3.72 ± 0.06	C_15_H_24_	97.51	1491	1454
49.19	*α*-Guaiene	7.29 ± 0.09	C_15_H_24_	97.45	1496	1473
49.4	*γ*-Muurolene	2.38 ± 0.03	C_15_H_24_	97.42	1501	1477
49.9	Germacrene D	4.1 ± 0.19	C_15_H_24_	97.28	1502	1481
50.85	Myristicin	9.41 ± 0.06	C_11_H_12_O_3_	98.73	1536	1519
51.12	*δ*-Cadinene	8.79 ± 0.19	C_15_H_24_	96.57	1543	1524
51.27	epi-cubebol	10.16 ± 0.44	C_15_H_26_O	94.99	1546	1515
56.39	*τ*-Cadinol	2.09 ± 0.13	C_15_H_26_O	96.79	1674	1640
56.96	*α*-Cadinol	3.6 ± 0.13	C_15_H_26_O	94.68	1689	1653
*Piper lanceifolium*						
22.86	Camphene	3.70 ± 0.08	C_10_H_16_	95.72	966	952
24.21	*β*-Pinene	9.25 ± 0.16	C_10_H_16_	96.18	991	979
25.68	*α*-Phellandrene	4.26 ± 0.06	C_10_H_16_	97.95	1018	1005
26.95	Limonene	14.98 ± 0.00	C_10_H_16_	98.26	1041	1018
27.17	*β*-Phellandrene	4.54 ± 0.06	C_10_H_16_	94.58	1045	1031
28.41	*γ*-Terpinene	9.27 ± 0.07	C_10_H_16_	98.38	1068	1060
37.91	Geraniol	1.02 ± 0.02	C_10_H_18_O	92.92	1251	1255
40.3	Bornyl acetate	1.25 ± 0.01	C_12_H_20_O_2_	96.74	1300	1285
43.3	Eugenol	2.23 ± 0.03	C_10_H_12_O_2_	97.02	1364	1357
45.08	Copaene	2.12 ± 0.02	C_15_H_24_	96.56	1403	1376
47.39	*β*-Caryophyllene	12.3 ± 0.04	C_15_H_24_	99.1	1455	1419
48.51	Cadina-3,5-diene	1.39 ± 0.02	C_15_H_24_	95.96	1481	1458
49.67	*γ*-Muurolene	3.00 ± 0.02	C_15_H_24_	95.83	1508	1477
52.19	Nerolidol	6.32 ± 0.06	C_15_H_26_O	96.22	1569	1564
54.92	Apiol	14.94 ± 0.35	C_12_H_14_O_4_	94.23	1637	1682
58.3	Farnesol	2.54 ± 0.00	C_15_H_26_O	98.1	1724	1713

RT, retention time (min); RA, relative abundance (%), calculated from GC peak areas without correction factors; MF, match factor (%), representing the similarity score obtained from mass spectral library comparison; RI, retention index. Compounds were identified by comparison with the NIST 17 Mass Spectral Library. The complete list of detected compounds is provided in the Supplementary Material Table S8.

**Table 2:** *p*-values from two-way ANOVA evaluating the effects of essential oils, dose, and their interaction on antibacterial inhibition.

Study’s Factors	Gram-Negative Bacteria		Gram-Positive Bacteria	
	*E. coli*	*S. enterica*	*E. faecalis*	*S. aureus*
EO	<0.0001	<0.0001	<0.0001	<0.0001
Dose	<0.0001	<0.0001	<0.0001	<0.0001
EO × Dose	<0.0001	<0.0001	<0.0001	<0.0001

Bacterial strains were *Escherichia coli* ATCC^®^ 25922™, *Salmonella enterica* subsp. *enterica serovar Typhimurium* ATCC^®^ 14028™, *Enterococcus faecalis* ATCC^®^ 29212™, and *Staphylococcus aureus* subsp. *aureus* ATCC^®^ 49476™.

**Table 3:** Dose–response parameters (LD_50_) of essential oils against four food-borne bacterial strains estimated using a log-logistic model.

Food-Borne Bacterial Strains	Essential oils	LD_50_ (%)	*p*	R^2^	RMSE
Gram-negative					
*Escherichia coli* (ATCC^®^ 25922™)	*Aloysia citrodora*	8.97 ± 1.66	<0.0001	0.87	3.67
	*Arracacia xanthorrhiza* cv. Yellow	37.70 ± 1.53	<0.0001	0.98	5.13
	*Arracacia xanthorrhiza* cv. Purple	49.17 ± 0.51	<0.0001	0.99	2.08
*Salmonella enterica* subsp. *enterica* (ATCC^®^ 14028™)	*Aloysia citrodora*	34.20 ± 7.78	<0.0001	0.76	19.11
	*Arracacia xanthorrhiza* cv. Yellow	29.55 ± 3.62	<0.0001	0.84	6.39
	*Arracacia xanthorrhiza* cv. Purple	94.26 ± 15.24	<0.0001	0.76	12.13
Gram-positive					
*Enterococcus faecalis* (ATCC^®^ 29212™)	*Aloysia citrodora*	28.71 ± 1.36	<0.0001	0.97	6.11
	*Arracacia xanthorrhiza* cv. Yellow	18.59 ± 2.66	<0.0001	0.85	8.38
	*Arracacia xanthorrhiza* cv. Purple	49.70 ± 0.30	<0.0001	0.99	1.95
	*Baccharis genistelloides*	21.29 ± 4.39	<0.0001	0.74	13.57
	*Piper acutifolium*	88.26 ± 7.37	<0.0001	0.89	8.10
	*Piper lanceifolium*	87.75 ± 11.50	<0.0001	0.81	11.25
*Staphylococcus aureus* subsp. *aureus* (ATCC^®^ 49476™)	*Aloysia citrodora*	27.06 ± 3.87	<0.0001	0.83	10.13
	*Arracacia xanthorrhiza* cv. Yellow	18.43 ± 3.99	<0.0001	0.72	13.24
	*Arracacia xanthorrhiza* cv. Purple	42.57 ± 0.95	<0.0001	0.99	2.85
	*Baccharis genistelloides*	91.09 ± 10.35	<0.0001	0.92	2.08
	*Piper acutifolium*	122.97 ± 16.31	<0.0001	0.84	7.55
	*Piper lanceifolium*	123.24 ± 24.22	<0.0001	0.85	2.99

LD_50_ values represent model-derived estimates of the concentrations (%) required to inhibit 50% of bacterial growth according to the fitted log-logistic model. Values above 100% indicate that 50% inhibition was not achieved within the experimentally evaluated concentration range and therefore represent extrapolated estimates associated with relatively low antibacterial activity. *p*-values correspond to the significance of the log-logistic dose–response model.

**Table 4:** Reported antibacterial activity of the major constituents identified in the evaluated essential oils.

Compound	Maximum Abundance (%)	EO Source	Reported Antibacterial Activity	References
Limonene	17.33	*A. citrodora*, *P. lanceifolium*	Reported antibacterial activity against *Escherichia coli*, *Staphylococcus aureus*, and other food-borne pathogens. Also exhibits antimicrobial and antibiofilm effects against uropathogenic *Klebsiella pneumoniae*, including NDM-1-producing strains.	[50,51,52]
Citronellol (β-citronellol)	15.06	*A. citrodora*	Antibacterial and antibiofilm activity against uropathogenic *K. pneumoniae*, including NDM-1-producing strains. Membrane permeability alterations have been proposed as one of its mechanisms of action.	[50,53]
Linalool	15.37	*P. acutifolium*, *A. xanthorrhiza*	Broad-spectrum antibacterial activity reported against Gram-positive and Gram-negative bacteria, including antibiofilm effects against *K. pneumoniae*. Proposed mechanisms include membrane disruption and interference with cellular metabolism.	[50,54,55]
trans-β-Ocimene	30.96	*A. xanthorrhiza*	Limited direct evidence is available for isolated trans-β-ocimene. However, antibacterial activity has been predicted against *Staphylococcus simulans* and *Streptococcus mutans*, and ocimene-rich essential oils have shown strong activity against resistant nosocomial pathogens, suggesting a potential contribution through additive or synergistic interactions with other terpenes.	[56,57,58]
Sabinene	21.46	*A. xanthorrhiza*	Antimicrobial activity reported against oral pathogens. In *Streptococcus mutans*, sabinene suppresses growth, biofilm formation, and adhesion through downregulation of virulence-associated genes.	[59,60]
β-Pinene	16.21	*A. xanthorrhiza*	Antibacterial activity reported against multiple bacterial species (MIC range 0.25–4.00 mg/mL). Synergistic interactions with other terpenes have been described, including combinations with β-caryophyllene, estragole, and ocimene.	[58,61]
β-Caryophyllene	24.92	Several oils	Antibacterial and antibiofilm activity has been reported against both Gram-positive and Gram-negative bacteria. Proposed mechanisms include inhibition of bacterial efflux pumps (QacA/B and NorA), enhancement of antibiotic efficacy, and interference with virulence-related factors such as the Esp surface protein of *Enterococcus faecalis*.	[50,62,63,64,65]
Apiol	14.94	*P. lanceifolium*	Limited evidence of antibacterial activity; reported in some studies against Salmonella and Vibrio spp., but inactive when tested as a pure compound (up to 200 μg/mL) against other bacterial strains. May require synergy with other EO components.	[66]
Myristicin	9.41	*P. acutifolium*	Antimicrobial and antibiofilm activity reported against several bacterial species, including *S. aureus*, *E. coli*, and *Micrococcus luteus*. Proposed mechanisms include membrane permeabilization and inhibition of biofilm formation. Synergistic or additive interactions with ampicillin have also been reported against *Salmonella* and *Vibrio* spp.	[66,67,68]

Note: EO source indicates the essential oil(s) evaluated in the present study, in which each compound was identified among the major constituents. The reported antibacterial activities correspond to findings from previous studies and are included to provide context for the potential contribution of these compounds to the antibacterial activity observed herein. Because essential oils are complex mixtures, the observed bioactivity is likely influenced by additive and/or synergistic interactions among multiple constituents rather than by any single compound alone.

**Table 5:** Botanical information and collection details of plant species used for essential oil extraction.

Scientific Name	Common Name	Collection Coordinates (WGS84)	Altitude (m a.s.l.)	Plant Part Used	Voucher Code
*Aloysia citrodora* Paláu	Lemon verbena, cedrón	6.240147° S, 77.880824° W	2190	Leaves and inflorescences	KUELA*P*-6731
*Arracacia xanthorrhiza* Bancr. cv. Yellow	Virraca, zanahoria amarilla, zacacha amarilla	6.223619° S, 77.622660° W	2525	Leaves	KUELA*P*-6732
*Arracacia xanthorrhiza* Bancr. cv. Purple	Virraca, zanahoria morada, zacacha morada	6.225439° S, 77.624474° W	2548	Leaves	KUELA*P*-6733
*Baccharis genistelloides* (Lam.) Pers.	Carqueja, tres esquinas	6.220847° S, 77.593626° W	2657	Leaves, stems, and flowers	KUELA*P*-6734
*Piper acutifolium* Ruiz & Pav.	Matico macho, matico	6.224259° S, 77.622447° W	2487	Leaves and inflorescences	KUELA*P*-6738
*Piper lanceifolium* Kunth	Cordoncillo alimondado, pepper vine	6.225364° S, 77.625805° W	2527	Leaves and inflorescences	KUELA*P*-6739

## Data Availability

The original contributions presented in this study are included in the article/Supplementary Material. Further inquiries can be directed to the corresponding authors.

## Supplementary Materials

The following supporting information can be downloaded at: https://www.mdpi.com/article/10.3390/ph19060951/s1, Table S1: Inhibition of food-borne pathogenic bacteria by six essential oils from plants in northeastern Peru; Table S2: Two-way ANOVA results for antibacterial inhibition; Table S3: Tukey HSD comparison among essential oils; Table S4: Tukey HSD comparison among doses; Table S5. Contributions (%) of variables to the principal components of the PCA; Table S6. Variable loadings for PCA components; Table S7. PCA scores of essential oils; Table S8. Chemical composition and relative abundance (%) of volatile compounds identified in the essential oils by GC–MS.
