# Supplementary material for: Chemical Composition and Antibacterial Activity Against Food-Borne Pathogens of Six Essential Oils from Plants in Northeastern Peru
Source: Pharmaceuticals (Basel). 2026 Jun 18;19(6):951. doi: 10.3390/ph19060951 (PMC13304477; doi:10.3390/ph19060951)
Supplement: Supplementary file 1 [file pharmaceuticals-19-00951-s001.zip › pharmaceuticals-4355828-supplementary.pdf]

Chemical composition and antibacterial activity against foodborne pathogens of six essential oils from plants in northeastern Peru

Laydy Mitsu Mena-Chacon [1,2], Krizia Pretell [1,3], Angel F. Huaman-Pilco [2], Yuriko Saavedra [3], Aline Camila Caetano [4], Diner Mori-Mestanza [1], Robin Oblitas-Delgado [2], Carlos A. Amasifuen-Guerra [5], Rocio Jara-Vilca [2], Roberth Esteve Iliquin-Fernandez [1,6], and Segundo Chávez-Quintana [6*]

[1] Escuela de Posgrado, Programa Doctoral en Ciencias para el Desarrollo Sustentable, Facultad de Ingeniería Zootecnista, Agronegocios, Biotecnología y Ciencias de Datos, Universidad Nacional Toribio Rodríguez de Mendoza de Amazonas, Chachapoyas, 01001, Perú
[2] Grupo de Investigación en Biopesticidas y Bioalternativas para la Protección Vegetal (BIOPEST), Instituto de Investigación para el Desarrollo Sustentable de Ceja de Selva, Universidad Nacional Toribio Rodríguez de Mendoza de Amazonas, Chachapoyas, Perú
[3] Inca Biotec S.A.C., Jr. Filipinas 212, Tumbes, Perú
[4] Grupo de Investigación en Hongos y Deterioro de Alimentos (GIHDA), Instituto de Investigación para el Desarrollo Sustentable de Ceja de Selva, Universidad Nacional Toribio Rodríguez de Mendoza de Amazonas, Chachapoyas, Perú
[5] Instituto de Investigación en Forestería y Ecosistemas Tropicales (INIFET), Universidad Nacional Toribio Rodríguez de Mendoza de Amazonas (UNTRM), Chachapoyas 01001, Perú
[6] Instituto de Investigación para el Desarrollo Sustentable de Ceja de Selva, Universidad Nacional Toribio Rodríguez de Mendoza de Amazonas, Chachapoyas, Perú
* Correspondence: laydy.mena@untrm.edu.pe (L.M.M.-C.); segundo.quintana@untrm.edu.pe (S.C.-Q.)

Supplementary Table S1. Inhibition of foodborne pathogenic bacteria by six essential oils from plants in northeastern Peru

**Essential oil**	Dose (%)	**Inhibition (%)**											
		E. coli **(-)**			***S. enterica*** **(-)**			***E. faecalis*** **(+)**			***S. aureus*** **(+)**		
		**A**	**B**	**C**	**A**	**B**	**C**	**A**	**B**	**C**	**A**	**B**	**C**
Piper lanceifolium	10	0.00	0.00	0.00	0.00	0.00	0.00	0.00	0.00	0.00	33.21	32.56	32.88
	30	0.00	0.00	0.00	0.00	0.00	0.00	0.00	0.00	0.00	35.44	36.23	35.84
	50	0.00	0.00	0.00	0.00	0.00	0.00	40.60	43.07	41.83	39.81	42.00	40.91
	100	0.00	0.00	0.00	0.00	0.00	0.00	50.52	50.52	50.52	53.81	47.81	50.81
Piper acutifolium	10	0.00	0.00	0.00	0.00	0.00	0.00	0.00	0.00	0.00	0.00	0.00	0.00
	30	0.00	0.00	0.00	0.00	0.00	0.00	0.00	0.00	0.00	0.00	0.00	0.00
	50	0.00	0.00	0.00	0.00	0.00	0.00	34.77	33.64	34.21	28.84	28.37	28.60
	100	0.00	0.00	0.00	0.00	0.00	0.00	51.59	54.39	52.99	38.60	39.53	39.07
Aloysia citrodora	10	55.87	50.28	53.07	27.46	28.79	28.12	0.00	0.00	0.00	34.70	33.21	33.95
	30	61.45	55.87	58.66	35.43	38.53	36.98	56.30	57.59	56.94	46.28	40.05	43.16
	50	67.04	72.63	69.83	67.76	68.64	68.20	75.98	79.18	77.58	61.95	59.77	60.86
	100	77.09	75.42	76.26	122.72	113.64	118.18	92.58	85.40	88.99	93.35	91.35	92.35
Arracacia xanthorrhiza cv. Yellow	10	0.00	0.00	0.00	36.36	35.87	36.12	42.06	40.37	41.21	42.56	41.86	42.21
	30	39.11	39.11	39.11	39.86	40.74	42.52	50.07	48.45	49.26	47.67	38.88	43.28
	50	67.04	68.44	67.74	65.32	64.35	64.84	76.09	74.52	75.31	84.42	84.84	84.63
	100	82.51	81.73	82.12	69.84	69.22	69.53	91.79	88.26	90.03	88.65	92.70	90.67
Arracacia xanthorrhiza cv. Purple	10	0.00	0.00	0.00	0.00	0.00	0.00	0.00	0.00	0.00	0.00	0.00	0.00
	30	0.00	0.00	0.00	0.00	0.00	0.00	0.00	0.00	0.00	34.37	33.95	34.16
	50	54.53	54.53	54.53	43.36	42.16	42.76	50.69	52.77	51.73	59.35	57.40	58.37
	100	94.97	97.77	96.37	46.46	47.03	46.74	101.33	94.54	97.93	83.72	84.65	84.19
Baccharis genistelloides	10	0.00	0.00	0.00	0.00	0.00	0.00	40.37	38.13	39.25	33.72	35.35	34.53
	30	0.00	0.00	0.00	0.00	0.00	0.00	48.22	49.07	48.79	38.23	37.91	38.14
	50	0.00	0.00	0.00	0.00	0.00	0.00	61.68	64.49	63.08	45.58	44.88	45.23
	100	0.00	0.00	0.00	0.00	0.00	0.00	102.34	103.74	103.04	51.63	52.56	52.09

Essential oil	Dose (%)	Inhibition (%)											
		***E. coli* (-)**			***S. enterica* (-)**			***E. faecalis* (+)**			***S. aureus* (+)**		
		**A**	**B**	**C**	**A**	**B**	**C**	**A**	**B**	**C**	**A**	**B**	**C**
**Control**		**Inhibition (mm)**											
Positive control		18.8			21.94			18			21.5		
		16.9			23			18			21		
		18			22.8			17.5			22		
Negative control		0			0			0			0		

Strains: Escherichia coli ATCC® 25922™, *Salmonella enterica* subsp. *enterica* ATCC® 14028™, *Enterococcus faecalis* ATCC® 29212™, and *Staphylococcus aureus* subsp. *aureus* ATCC® 49476™. Positive controls included vancomycin (30 µg) for *E. faecalis*, amoxicillin–clavulanic acid (30 µg) for *E. coli*, and florfenicol (30 µg) for *S. enterica* and *S. aureus*. DMSO (100 %) was used as a negative control.

Supplementary Table S2. Two-way ANOVA results for antibacterial inhibition

Bacteria	Source of variation	df	F	p-value
Escherichia coli	Essential oil	5	8819.26	<0.0001
	Dose	3	3725.15	<0.0001
	EO × Dose	15	1202.54	<0.0001
Salmonella enterica	Essential oil	5	8907.21	<0.0001
	Dose	3	3004.67	<0.0001
	EO × Dose	15	824.76	<0.0001
Enterococcus faecalis	Essential oil	5	2644.28	<0.0001
	Dose	3	9710.98	<0.0001
	EO × Dose	15	329.59	<0.0001
Staphylococcus aureus	Essential oil	5	1626.19	<0.0001
	Dose	3	3539.18	<0.0001
	EO × Dose	15	217.05	<0.0001

Supplementary Table S2. Summary of two-way ANOVA evaluating the effects of essential oil (EO), dose, and their interaction on antibacterial inhibition for each bacterial strain. *Escherichia coli* ATCC® 25922™, *Salmonella enterica* subsp. *enterica* ATCC® 14028™, *Enterococcus faecalis* ATCC® 29212™, and *Staphylococcus aureus* subsp. *aureus* ATCC® 49476™.

Supplementary Table S3. Tukey HSD comparison among essential oils

Bacteria	Essential oil	Mean inhibition (%)	Group
Escherichia coli	*Aloysia citrodora*	64.46	A
	A. xanthorrhiza cv. Yellow	47.24	B
	A. xanthorrhiza cv. Purple	37.72	C
	Piper acutifolium	0	D
	Piper lanceifolium	0	D
	Baccharis genistelloides	0	D
Salmonella enterica	*Aloysia citrodora*	62.87	A
	A. xanthorrhiza cv. Yellow	52.88	B
	A. xanthorrhiza cv. Purple	22.38	C
	Piper lanceifolium	0	D
	Piper acutifolium	0	D
	Baccharis genistelloides	0	D
Enterococcus faecalis	*A. xanthorrhiza* cv. Yellow	63.95	A
	Baccharis genistelloides	63.52	A
	Aloysia citrodora	55.88	B
	A. xanthorrhiza cv. Purple	37.42	C
	Piper lanceifolium	23.09	D
	Piper acutifolium	21.8	D
Staphylococcus aureus	*A. xanthorrhiza* cv. Yellow	65.2	A
	Aloysia citrodora	57.58	B
	A. xanthorrhiza cv. Purple	44.18	C
	Baccharis genistelloides	42.49	C
	Piper lanceifolium	40.11	D
	Piper acutifolium	16.92	E

Multiple comparison of antibacterial inhibition among essential oils according to Tukey's HSD test ($\alpha = 0.05$). Means sharing the same letter are not significantly different. *Escherichia coli* ATCC® 25922™, *Salmonella*

enterica subsp. *enterica* ATCC® 14028™, *Enterococcus faecalis* ATCC® 29212™, and *Staphylococcus aureus* subsp. *aureus* ATCC® 49476™.

Supplementary Table S4. Tukey HSD comparison among doses

Bacteria	Dose (%)	Mean inhibition (%)	Group
Escherichia coli	100	42.46	A
	50	32.02	B
	30	16.29	C
	10	8.85	D
Salmonella enterica	100	39.08	A
	50	29.3	B
	30	13	C
	10	10.71	D
Enterococcus faecalis	100	80.58	A
	50	57.29	B
	30	25.82	C
	10	13.41	D
Staphylococcus aureus	100	68.2	A
	50	53.1	B
	30	32.42	C
	10	23.93	D

Multiple comparison of antibacterial inhibition among doses according to Tukey's HSD test ($\alpha = 0.05$). Means sharing the same letter are not significantly different. *Escherichia coli* ATCC® 25922™, *Salmonella enterica* subsp. *enterica* ATCC® 14028™, *Enterococcus faecalis* ATCC® 29212™, and *Staphylococcus aureus* subsp. *aureus* ATCC® 49476™.

Supplementary Table S5. Contributions (%) of variables to the principal components of the PCA

Variable	PC1 (%)	PC2 (%)	PC3 (%)	PC4 (%)	PC5 (%)
Aliphatic alcohol	7.38	7.73	5.93	5.57	11.26
Aliphatic aldehyde	9.69	6.98	1.51	6.34	0.17
Aliphatic ketone	7.38	7.73	5.93	5.57	11.26
Diterpene hydrocarbon	3.18	10.43	12.84	3.24	2.53
Ester	3.56	11.66	10.18	4.09	3.62
Phenylpropanoid	3.56	8.56	10.98	30.32	1.29
Monoterpene hydrocarbon	1.77	17.61	7.05	0.57	4.36
Oxygenated monoterpene	10.22	1.31	6.67	14.38	1.71
Sesquiterpene hydrocarbon	5.3	16.62	0.02	0.03	5.34
Oxygenated sesquiterpene	6.76	4.99	9.08	22.29	0.2
E. coli	11.7	1.61	3.81	5.35	6.6
S. enterica	13.88	0.67	0.09	0.03	29.2
E. faecalis	2.76	3.92	22.75	0.62	2.05
S. aureus	12.87	0.18	3.15	1.6	19.71

Percentage contribution of chemical families and antibacterial activity variables to the principal components obtained in the PCA analysis. *Escherichia coli* ATCC® 25922™, *Salmonella enterica* subsp. *enterica* ATCC® 14028™, *Enterococcus faecalis* ATCC® 29212™, and *Staphylococcus aureus* subsp. *aureus* ATCC® 49476™.

Supplementary Table S6. Variable loadings for PCA components

Variable	PC1	PC2	PC3	PC4	PC5
Aliphatic alcohol	0.713	0.542	-0.414	0.143	-0.077
Aliphatic aldehyde	0.817	0.515	-0.209	0.153	-0.009
Aliphatic ketone	0.713	0.542	-0.414	0.143	-0.077
Diterpene hydrocarbon	-0.468	0.63	0.609	0.109	-0.036
Ester	-0.495	0.666	0.543	0.123	0.044
Phenylpropanoid	-0.495	-0.57	-0.564	0.334	0.026
Monoterpene hydrocarbon	0.349	-0.818	0.451	0.046	-0.048
Oxygenated monoterpene	0.838	0.223	-0.439	-0.23	0.03
Sesquiterpene hydrocarbon	-0.604	0.795	-0.021	0.01	0.053
Oxygenated sesquiterpene	-0.682	0.435	-0.512	-0.286	-0.01
E. coli	0.897	-0.247	0.332	-0.14	-0.059
S. enterica	0.978	0.16	-0.052	0.011	0.125
E. faecalis	0.436	0.386	0.811	-0.048	-0.033
S. aureus	0.941	-0.083	0.302	0.077	0.102

Variable loadings associated with the principal components of the PCA analysis integrating chemical composition and antibacterial activity variables. *Escherichia coli* ATCC® 25922™, *Salmonella enterica* subsp. *enterica* ATCC® 14028™, *Enterococcus faecalis* ATCC® 29212™, and *Staphylococcus aureus* subsp. *aureus* ATCC® 49476™.

Supplementary Table S7. PCA scores of essential oils

Essential oil	PC1	PC2	PC3	PC4	PC5
Aloysia citriodora	4.182	2.364	-1.575	0.194	-0.039
Arracacia xanthorrhiza cv. Purple	1.545	-1.762	1.775	-0.283	-0.365
Arracacia xanthorrhiza cv. Yellow	1.681	-1.638	1.148	-0.115	0.422
Baccharis genistelloides	-2.871	2.898	2.082	0.177	0.022
Piper acutifolium	-2.46	-0.122	-2.019	-0.998	0.005
Piper lanceifolium	-2.077	-1.74	-1.41	1.025	-0.045

PCA scores of essential oils based on chemical families and antibacterial activity variables.

Supplementary Table S8. Chemical composition and relative abundance (%) of volatile compounds identified in the essential oils by GC–MS

RT (min)	Compound name	RA (%)	Formula	MF (%)	Experimental RI	Library RI
Aloysia citrodora						
21.77	Tricyclene	1.19 ± 0.06	$C_{10}H_{16}$	98.59	946	890
23.48	1-Octen-3-ol	0.91 ± 0.05	$C_8H_{16}O$	95.59	977	980
23.77	Sulcatone	5.53 ± 0.11	$C_8H_{14}O$	91.96	983	986
24.19	*β*-Myrcene	1.30 ± 0.04	$C_{10}H_{16}$	96.11	990	991
24.41	3-Octanol	0.38 ± 0.07	$C_8H_{18}O$	94.00	994	994
25.67	*α*-Phellandrene	0.77 ± 0.00	$C_{10}H_{16}$	91.60	1018	1005
**26.95**	**Limonene**	**17.33 ± 0.18**	$\mathbf{C_{10}H_{16}}$	**98.07**	**1041**	**1018**
27.72	5-Heptenal, 2,6-dimethyl-	1.27 ± 0.06	$C_9H_{16}O$	92.65	1055	1052
28.40	*γ*-Terpinene	0.18 ± 0.01	$C_{10}H_{16}$	91.91	1068	1060

RT (min)	Compound name	RA (%)	Formula	MF (%)	Experimental RI	Library RI
29.15	Fenchol	0.68 ± 0.00	$C_{10}H_{18}O$	97.35	1082	1075
29.99	Terpinolene	0.19 ± 0.01	$C_{10}H_{16}$	94.74	1097	1088
30.21	Linalool	1.06 ± 0.01	$C_{10}H_{18}O$	97.24	1101	1099
30.43	Nonanal	0.84 ± 0.01	$C_9H_{18}O$	96.89	1105	1104
33.77	1-Nonanol	0.07 ± 0.00	$C_9H_{20}O$	91.76	1169	1173
34.39	Neral	2.98 ± 0.00	$C_{10}H_{16}O$	96.85	1181	1184
35.22	Terpinen-4-ol	0.41 ± 0.00	$C_{10}H_{18}O$	90.60	1197	1177
35.82	α-Terpineol	1.97 ± 0.01	$C_{10}H_{18}O$	97.34	1209	1189
36.70	Citronellol	15.06 ± 0.01	$C_{10}H_{20}O$	97.90	1227	1220
36.84	Nerol	2.76 ± 0.02	$C_{10}H_{18}O$	98.34	1230	1228
37.93	Geraniol	3.26 ± 0.02	$C_{10}H_{18}O$	98.76	1252	1255
39.96	2-Undecanone	1.25 ± 0.01	$C_{11}H_{22}O$	92.51	1293	1294
40.91	Dihydrocarvyl acetate	0.38 ± 0.01	$C_{12}H_{20}O_2$	95.03	1313	1306
42.50	Citronellyl acetate	0.39 ± 0.01	$C_{12}H_{22}O_2$	96.48	1347	1354
43.31	Eugenol	0.26 ± 0.00	$C_{10}H_{12}O_2$	92.95	1364	1357
43.82	Geranyl acetate	4.64 ± 0.04	$C_{12}H_{20}O_2$	98.50	1375	1382
45.09	Copaene	0.89 ± 0.02	$C_{15}H_{24}$	96.48	1403	1376
46.64	Citronellyl propionate	0.44 ± 0.01	$C_{13}H_{24}O_2$	96.58	1438	1445
47.39	β-Caryophyllene	8.09 ± 0.11	$C_{15}H_{24}$	93.91	1455	1419
47.91	Geranyl propionate	2.87 ± 0.09	$C_{13}H_{22}O_2$	92.81	1467	1475
49.17	Cuparene	10.11 ± 0.07	$C_{15}H_{22}$	96.27	1496	1483
49.70	Germacrene D	3.28 ± 0.08	$C_{15}H_{24}$	98.26	1509	1495
50.35	δ-Elemene	2.28 ± 0.01	$C_{15}H_{24}$	95.10	1524	1514
52.19	Nerolidol	2.95 ± 0.09	$C_{15}H_{26}O$	97.21	1569	1564
54.49	Caryophyllene oxide	4.03 ± 0.16	$C_{15}H_{24}O$	94.79	1626	1581
***Arracacia xanthorrhiza* cv. Yellow**						
21.10	α-Pinene	1.24 ± 0.02	$C_{10}H_{16}$	94.71	934	937
23.83	Sabinene	21.46 ± 0.04	$C_{10}H_{16}$	95.26	984	974
24.39	β-Pinene	16.21 ± 0.01	$C_{10}H_{16}$	94.58	994	979
24.89	Octanal	0.50 ± 0.01	$C_8H_{16}O$	98.95	1003	1003
25.68	α-Phellandrene	0.25 ± 0.00	$C_{10}H_{16}$	94.28	1018	1005
26.63	*p*-Cymene	2.72 ± 0.00	$C_{10}H_{14}$	97.31	1035	1025
**27.33**	**trans-β-Ocimene**	**30.96 ± 0.44**	$\mathbf{C_{10}H_{16}}$	**96.50**	**1048**	**1049**
28.40	γ-Terpinene	0.83 ± 0.04	$C_{10}H_{16}$	94.38	1068	1060
29.99	Terpinolene	0.76 ± 0.02	$C_{10}H_{16}$	93.23	1097	1088
30.21	Linalool	5.55 ± 0.35	$C_{10}H_{18}O$	97.47	1101	1099
31.94	trans-Carveol	0.95 ± 0.01	$C_{10}H_{16}O$	97.46	1134	1123
34.49	Pinocarvone	1.10 ± 0.02	$C_{10}H_{14}O$	95.36	1183	1171
35.21	α-Terpineol	1.14 ± 0.06	$C_{10}H_{18}O$	94.77	1197	1182
35.80	Estragole	2.89 ± 0.15	$C_{10}H_{12}O$	97.94	1209	1196
36.89	Carveol	1.68 ± 0.06	$C_{10}H_{16}O$	98.08	1231	1229
38.31	Carvone	1.27 ± 0.09	$C_{10}H_{14}O$	97.32	1259	1242
40.71	trans-Pinocarvyl acetate	1.19 ± 0.11	$C_{12}H_{18}O_2$	95.79	1309	1297
42.07	trans-Carveyl acetate	1.01 ± 0.16	$C_{12}H_{18}O_2$	97.00	1338	1337
45.01	Methyleugenol	1.36 ± 0.28	$C_{11}H_{14}O_2$	93.52	1401	1402
45.61	β-Bourbonene	1.10 ± 0.19	$C_{15}H_{24}$	95.05	1415	1384
47.37	β-Caryophyllene	1.29 ± 0.23	$C_{15}H_{24}$	96.54	1455	1419

RT (min)	Compound name	RA (%)	Formula	MF (%)	Experimental RI	Library RI
48.95	Humulene	0.33 ± 0.09	$C_{15}H_{24}$	91.06	1491	1454
49.95	Germacrene D	3.73 ± 0.72	$C_{15}H_{24}$	94.34	1515	1481
56.93	*α*-Cadinol	0.46 ± 0.16	$C_{15}H_{26}O$	93.03	1688	1653
***Arracacia xanthorrhiza* cv. Purple**						
23.83	Sabinene	20.94 ± 0.48	$C_{10}H_{16}$	95.23	984	974
24.39	*β*-Pinene	14.16 ± 0.39	$C_{10}H_{16}$	94.45	994	979
24.89	Octanal	0.38 ± 0.02	$C_8H_{16}O$	99.05	1003	1003
25.67	*α*-Phellandrene	0.22 ± 0.00	$C_{10}H_{16}$	96.49	1018	1005
26.24	Terpinene	0.19 ± 0.01	$C_{10}H_{16}$	95.14	1028	1017
26.63	*p*-Cymene	2.04 ± 0.04	$C_{10}H_{14}$	97.41	1035	1025
26.79	trans-*β*-Ocimene	10.15 ± 0.12	$C_{10}H_{16}$	98.84	1038	1049
**27.34**	***β*-Ocimene**	**30.05 ± 1.13**	$\mathbf{C_{10}H_{16}}$	**98.60**	1048	1037
28.40	*γ*-Terpinene	1.86 ± 0.13	$C_{10}H_{16}$	98.55	1068	1060
30.21	Linalool	8.47 ± 0.03	$C_{10}H_{18}O$	97.47	1101	1099
31.94	trans-Carveol	0.46 ± 0.02	$C_{10}H_{16}O$	97.28	1134	1123
35.21	Terpinen-4-ol	2.04 ± 0.03	$C_{10}H_{18}O$	93.33	1197	1182
35.80	Estragole	1.93 ± 0.00	$C_{10}H_{12}O$	98.95	1209	1196
36.89	cis-Carveol	0.29 ± 0.01	$C_{10}H_{16}O$	97.21	1231	1229
37.58	Carveol	0.39 ± 0.01	$C_{10}H_{16}O$	93.79	1245	1219
38.31	Carvone	0.51 ± 0.06	$C_{10}H_{14}O$	94.76	1260	1242
40.07	Perillaldehyde	0.32 ± 0.00	$C_{10}H_{14}O$	97.38	1295	1272
40.71	trans-Pinocarvyl acetate	0.88 ± 0.01	$C_{12}H_{18}O_2$	96.85	1309	1297
42.07	trans-Carveyl acetate	0.68 ± 0.03	$C_{12}H_{18}O_2$	97.27	1338	1337
43.81	Geranyl acetate	0.32 ± 0.01	$C_{12}H_{20}O_2$	95.67	1375	1382
45.61	(-)-*β*-Bourbonene	0.96 ± 0.00	$C_{15}H_{24}$	97.14	1415	1384
46.86	Perillyl acetate	0.09 ± 0.00	$C_{12}H_{18}O_2$	92.68	1443	1436
47.14	*β*-Copaene	0.15 ± 0.00	$C_{15}H_{24}$	94.53	1450	1421
47.37	*β*-Caryophyllene	1.45 ± 0.01	$C_{15}H_{24}$	98.18	1455	1419
50.83	Myristicin	0.22 ± 0.01	$C_{11}H_{12}O_3$	96.66	1536	1519
62.49	Neophytadiene	0.38 ± 0.01	$C_{20}H_{38}$	95.79	1837	1837
Baccharis genistelloides						
24.20	*β*-Myrcene	1.62 ± 0.01	$C_{10}H_{16}$	94.09	990	991
25.67	*α*-Phellandrene	1.23 ± 0.00	$C_{10}H_{16}$	97.64	1017	1005
26.22	Terpinene	0.20 ± 0.00	$C_{10}H_{16}$	93.99	1028	1017
26.65	trans-*β*-Ocimene	1.43 ± 0.00	$C_{10}H_{16}$	97.79	1035	1049
26.93	Limonene	6.82 ± 0.10	$C_{10}H_{16}$	98.25	1041	1018
27.16	*β*-Phellandrene	3.05 ± 0.01	$C_{10}H_{16}$	94.90	1045	1031
28.39	*γ*-Terpinene	0.56 ± 0.00	$C_{10}H_{16}$	98.09	1068	1060
29.98	*p*-Mentha-1,8-diene isomer	0.73 ± 0.00	$C_{10}H_{16}$	98.31	1097	1088
30.20	Linalool	0.89 ± 0.01	$C_{10}H_{18}O$	97.43	1101	1099
30.42	Nonanal	0.15 ± 0.00	$C_9H_{18}O$	97.39	1105	1104
32.62	Isobutyl hexanoate	0.31 ± 0.01	$C_{10}H_{20}O_2$	97.74	1147	1149
34.69	Hexanoic acid, butyl ester	0.51 ± 0.01	$C_{10}H_{20}O_2$	96.34	1187	1189
35.21	Terpinen-4-ol	0.42 ± 0.02	$C_{10}H_{18}O$	94.57	1197	1177
36.16	Safranal	0.16 ± 0.01	$C_{10}H_{14}O$	91.51	1216	1201
36.85	cis-3-Hexenyl-*α*-methylbutyrate	0.09 ± 0.00	$C_{11}H_{20}O_2$	95.03	1230	1234
37.06	n-Valeric acid cis-3-hexenyl ester	0.66 ± 0.00	$C_{11}H_{20}O_2$	95.19	1234	1237

RT (min)	Compound name	RA (%)	Formula	MF (%)	Experimental RI	Library RI
37.73	Isopentyl hexanoate	0.27 ± 0.00	$C_{11}H_{22}O_2$	94.81	1248	1252
37.85	2-Methylbutyl hexanoate	2.17 ± 0.01	$C_{11}H_{22}O_2$	97.45	1250	1247
38.23	Hexanoic acid, 4-pentenyl ester	1.11 ± 0.03	$C_{11}H_{20}O_2$	93.84	1258	1272
38.63	2-Decenal, (Z)-	0.17 ± 0.01	$C_{10}H_{18}O$	95.31	1266	1252
39.77	Prenyl hexanoate	0.06 ± 0.01	$C_{11}H_{20}O_2$	90.43	1289	1284
40.30	Bornyl acetate	0.40 ± 0.01	$C_{12}H_{20}O_2$	96.23	1300	1285
40.70	trans-Pinocarvyl acetate	0.23 ± 0.00	$C_{12}H_{18}O_2$	91.20	1309	1297
41.99	Myrtenyl acetate	0.21 ± 0.01	$C_{12}H_{18}O_2$	92.36	1336	1327
43.47	α-Cubebene	0.71 ± 0.02	$C_{15}H_{24}$	97.73	1368	1351
44.17	Hexanoic acid, hexyl ester	0.41 ± 0.02	$C_{12}H_{24}O_2$	96.32	1383	1384
44.79	Ylangene	1.21 ± 0.09	$C_{15}H_{24}$	94.76	1396	1372
45.08	Copaene	5.02 ± 0.11	$C_{15}H_{24}$	96.66	1403	1376
46.05	Shisool acetate	0.24 ± 0.01	$C_{12}H_{20}O_2$	90.73	1425	1419
**47.40**	**β-Caryophyllene**	**24.92 ± 0.46**	**$C_{15}H_{24}$**	**99.10**	1456	1419
49.42	γ-Muurolene	13.3 ± 0.10	$C_{15}H_{24}$	97.61	1502	1477
49.64	Naphthalene, 1,2,4a,5,6,8a-hexahydro-4,7-dimethyl-1-(1-methylethyl)-	8.21 ± 0.19	$C_{15}H_{24}$	95.01	1507	1485
50.33	α-Muurolene	4.64 ± 0.07	$C_{15}H_{24}$	97.04	1524	1499
52.18	Nerolidol	0.97 ± 0.04	$C_{15}H_{26}O$	97.82	1568	1564
55.80	Isospathulenol	1.41 ± 0.03	$C_{15}H_{24}O$	90.45	1659	1638
56.36	τ-Cadinol	2.88 ± 0.07	$C_{15}H_{26}O$	97.14	1673	1640
56.95	α-Cadinol	7.67 ± 0.08	$C_{15}H_{26}O$	97.52	1688	1653
61.02	Benzyl Benzoate	1.55 ± 0.09	$C_{14}H_{12}O_2$	98.90	1798	1762
62.51	Neophytadiene	3.41 ± 0.33	$C_{20}H_{38}$	95.18	1837	1837
Piper acutifolium						
21.77	α-Pinene	0.59 ± 0.01	$C_{10}H_{16}$	97.65	946	937
24.19	β-Myrcene	0.82 ± 0.04	$C_{10}H_{16}$	93.80	990	991
24.38	β-Pinene	0.35 ± 0.02	$C_{10}H_{16}$	93.41	994	979
25.67	α-Phellandrene	0.1 ± 0.01	$C_{10}H_{16}$	93.65	1017	1005
26.66	trans-β-Ocimene	12.63 ± 0.6	$C_{10}H_{16}$	98.56	1036	1049
28.40	γ-Terpinene	1.92 ± 0.00	$C_{10}H_{16}$	94.30	1068	1060
29.99	Terpinolene	3.04 ± 0.12	$C_{10}H_{16}$	96.63	1097	1088
**30.22**	**Linalool**	**15.37 ± 0.52**	**$C_{10}H_{18}O$**	**97.93**	1101	1099
33.78	(+)-2-Bornanone	0.41 ± 0.02	$C_{10}H_{16}O$	97.25	1169	1144
37.37	Prenyl tiglate	0.08 ± 0.00	$C_{10}H_{16}O_2$	92.47	1240	1247
37.59	Neral	0.1 ± 0.01	$C_{10}H_{16}O$	92.30	1245	1240
38.14	Pulegone	0.65 ± 0.02	$C_{10}H_{16}O$	96.95	1256	1237
38.93	Geranial	0.19 ± 0.00	$C_{10}H_{16}O$	91.24	1272	1270
40.24	Anethole	0.33 ± 0.01	$C_{10}H_{12}O$	95.52	1299	1286
43.47	α-Cubebene	0.74 ± 0.01	$C_{15}H_{24}$	96.90	1368	1351
44.80	Ylangene	0.15 ± 0.00	$C_{15}H_{24}$	94.31	1396	1372
45.08	Copaene	2.86 ± 0.01	$C_{15}H_{24}$	97.09	1403	1376
47.38	β-Caryophyllene	8.12 ± 0.03	$C_{15}H_{24}$	99.07	1455	1419
48.96	Humulene	3.72 ± 0.06	$C_{15}H_{24}$	97.51	1491	1454
49.19	α-Guaiene	7.29 ± 0.09	$C_{15}H_{24}$	97.45	1496	1473
49.40	γ-Muurolene	2.38 ± 0.03	$C_{15}H_{24}$	97.42	1501	1477
49.90	Germacrene D	4.1 ± 0.19	$C_{15}H_{24}$	97.28	1502	1481

RT (min)	Compound name	RA (%)	Formula	MF (%)	Experimental RI	Library RI
50.85	Myristicin	9.41 ± 0.06	$C_{11}H_{12}O_3$	98.73	1536	1519
51.12	δ-Cadinene	8.79 ± 0.19	$C_{15}H_{24}$	96.57	1543	1524
51.27	epi-cubebol	10.16 ± 0.44	$C_{15}H_{26}O$	94.99	1546	1515
56.39	τ-Cadinol	2.09 ± 0.13	$C_{15}H_{26}O$	96.79	1674	1640
56.96	α-Cadinol	3.6 ± 0.13	$C_{15}H_{26}O$	94.68	1689	1653
Piper lanceifolium						
21.11	Sabinene	0.42 ± 0.01	$C_{10}H_{16}$	97.87	934	929
22.86	Camphene	3.70 ± 0.08	$C_{10}H_{16}$	95.72	966	952
24.21	β-Pinene	9.25 ± 0.16	$C_{10}H_{16}$	96.18	991	979
24.90	Octanal	0.09 ± 0.00	$C_8H_{16}O$	97.06	1003	1003
25.68	α-Phellandrene	4.26 ± 0.06	$C_{10}H_{16}$	97.95	1018	1005
**26.95**	**Limonene**	**14.98 ± 0.00**	$\mathbf{C_{10}H_{16}}$	**98.26**	**1041**	**1018**
27.17	β-Phellandrene	4.54 ± 0.06	$C_{10}H_{16}$	94.58	1045	1031
27.32	β-Ocimene	0.5 ± 0.01	$C_{10}H_{16}$	93.42	1048	1037
28.41	γ-Terpinene	9.27 ± 0.07	$C_{10}H_{16}$	98.38	1068	1060
30.21	Linalool	0.75 ± 0.03	$C_{10}H_{18}O$	91.75	1101	1099
31.46	1,3,8-*p*-Menthatriene	0.11 ± 0.00	$C_{10}H_{14}$	93.83	1125	1119
32.04	Fenchol	0.3 ± 0.00	$C_{10}H_{18}O$	97.63	1136	1113
33.78	(+)-2-Bornanone	0.09 ± 0.01	$C_{10}H_{16}O$	90.86	1169	1144
34.97	endo-Borneol	0.86 ± 0.01	$C_{10}H_{18}O$	97.95	1192	1167
36.81	Nerol	0.21 ± 0.00	$C_{10}H_{18}O$	94.30	1229	1228
37.91	Geraniol	1.02 ± 0.02	$C_{10}H_{18}O$	92.92	1251	1255
40.30	Bornyl acetate	1.25 ± 0.01	$C_{12}H_{20}O_2$	96.74	1300	1285
43.30	Eugenol	2.23 ± 0.03	$C_{10}H_{12}O_2$	97.02	1364	1357
43.47	α-Cubebene	0.22 ± 0.00	$C_{15}H_{24}$	93.87	1368	1351
43.81	Geranyl acetate	0.85 ± 0.02	$C_{12}H_{20}O_2$	97.80	1375	1382
45.08	Copaene	2.12 ± 0.02	$C_{15}H_{24}$	96.56	1403	1376
45.49	β-Copaene	0.48 ± 0.01	$C_{15}H_{24}$	96.25	1412	1432
47.39	β-Caryophyllene	12.3 ± 0.04	$C_{15}H_{24}$	99.10	1455	1419
48.51	Cadina-3,5-diene	1.39 ± 0.02	$C_{15}H_{24}$	95.96	1481	1458
49.67	γ-Muurolene	3 ± 0.02	$C_{15}H_{24}$	95.83	1508	1477
50.84	Myristicin	0.97 ± 0.02	$C_{11}H_{12}O_3$	96.28	1536	1519
51.11	δ-Cadinene	0.81 ± 0.04	$C_{15}H_{24}$	93.66	1543	1524
51.87	Cubenene	0.25 ± 0.01	C15H24	94.11	1561	1532
52.19	Nerolidol	6.32 ± 0.06	$C_{15}H_{26}O$	96.22	1569	1564
54.92	Apiol	14.94 ± 0.35	$C_{12}H_{14}O_4$	94.23	1637	1682
58.30	Farnesol	2.54 ± 0.00	$C_{15}H_{26}O$	98.10	1724	1713

RT, retention time (min); RA, relative abundance (%), calculated from GC peak areas without correction factors; MF, match factor (%), representing the similarity score obtained from mass spectral library comparison; RI, retention index. Compounds were identified by comparison with the NIST 17 Mass Spectral Library.
